# Hybrid-sport participation is associated with gut microbiota composition: an exploratory longitudinal study

**DOI:** 10.3389/fspor.2026.1868776

**Published:** 2026-08-06

**Authors:** Manuele Biazzo, Simon De Jaegher, Lucrezia Morganti, Evangelos Kirithras, Maria D’Aguanno, Christine Podrini

**Affiliations:** 1 The BioArte Ltd., Life Science Park, San Gwann, Malta; 2 Laboratory of Molecular Microbiology and Biotechnology (LAMMB), Department of Medical Biotechnologies, University of Siena, Siena, Italy

**Keywords:** athletic performance, exercise modality, full-length 16S rRNA gene sequencing, gut microbiota, microbiome-targeted interventions, shotgun metagenomics, species-level resolution

## Abstract

Hybrid sports combine high-intensity resistance exercise with sustained aerobic demands, yet their association with gut microbiota composition and response to dietary or microbiota-targeted interventions remains unclear. Hybrid sports athletes have rarely been examined in human gut microbiome studies, which have largely focused on endurance disciplines. We investigated whether the combined study-group characteristics, including exercise modality, dietary intervention, and microbiota-targeted supplementation, were associated with differences in gut microbiota composition Thirty-eight adults were recruited into three groups: Hyrox® athletes (dietary intervention plus microbiota-targeted supplementation), Muay Thai fighters (dietary intervention), and sedentary adults (no intervention). Stool samples were collected at from all participants and at follow-up from a subset after the three-month study period. Full-length 16S rRNA gene sequencing was performed for all samples (*n* = 38), while paired shotgun metagenomic sequencing was conducted in 15 participants with complete paired samples. Alpha diversity did not differ significantly between study groups or over time. Beta diversity analysis identified sex as the strongest determinant of microbial community structure, whereas study group showed a modest association. Differential abundance analyses identified study-group-associated differences in selected taxa, including lower Enterococcaceae abundance in Hyrox® athletes and differences in *Dialister hominis*, *Dialister massiliensis*, and *Succiniclasticum ruminis*. Paired differential-abundance analyses accounting for repeated measurements identified a limited number of significant species- and family-level taxa, including subgroup-specific changes in *Dialister succinatiphilus*, *Megasphaera elsdenii*, *Clostridium herbivorans*, and *Vampirovibrio chlorellavorus*. Exploratory shotgun metagenomic analyses performed in a subset did not identify statistically significant gene- or pathway-level differences after multiple-testing correction, although descriptive differences were observed in selected pathways. Together, these findings suggest that the study-group characteristics were associated with fine-scale differences in gut microbiota composition. No significant large-scale changes in community-level microbial diversity were detected over the study period, whereas paired differential-abundance analyses in participants with complete follow-up samples identified a limited number of significant taxon-level changes. Because exercise modality, dietary intervention, and microbiota-targeted supplementation differed simultaneously between study groups, these findings should be interpreted as observational and hypothesis-generating rather than evidence of independent effects of exercise modality. Validation in larger controlled longitudinal studies is therefore required.

## 1 Introduction

Human gut microbiota is a dynamic and complex ecosystem that plays a central role in host metabolism, immune regulation, and inflammatory homeostasis, and maintenance of intestinal barrier integrity. In physically active individuals, these functions are particularly relevant because the gut microbiota has been associated with nutrient utilization, short-chain fatty acid production, immune regulation, recovery from exercise-induced physiological stress, and adaptation to training. Its composition and functional potential are shaped by multiple lifestyle and host-associated factors, including diet, physical activity, age, and body composition. Controlled human studies have demonstrated that dietary changes can induce rapid and reproducible alterations in gut microbiota composition and metabolic activity, highlighting the responsiveness of the microbiome to lifestyle modulation (1). More broadly, the gut microbiota has been implicated in metabolic health, chronic disease risk, and host physiological regulation across the lifespan (2).

Physical activity has emerged as an important modulator of gut microbiota composition. Early work comparing elite athletes with sedentary controls reported increased microbial diversity and distinct taxonomic profiles in the former group (3). Subsequent studies have confirmed that professional athletes exhibit gut microbiota compositions that differ from those of sedentary individuals, even when accounting for dietary intake, suggesting that exercise itself contributes to microbiota variation (4). Together, these findings indicate that habitual training status is independently associated with variation in the gut microbiome. Nevertheless, interpretation of these associations remains challenging because exercise and other lifestyle factors, including dietary intake, body composition, and supplement use, frequently coexist and may jointly influence gut microbiota composition. Beyond the general effects of physical activity, growing evidence suggests that gut microbiota characteristics may vary across athletic populations, reflecting differences in training load, intensity, and metabolic demands. Multi-omics analyses have identified specific microbial taxa and functional features associated with endurance exercise capacity and performance-related phenotypes (5). However, most existing research has focused on endurance-based disciplines, and athletes engaged in hybrid or multimodal training that combines endurance and resistance exercise remain largely unexplored in human gut microbiome studies.

Interpretation of athlete microbiome studies is complicated by the presence of multiple overlapping lifestyle and physiological factors that may influence gut microbial composition independently of exercise modality. Dietary intake, energy availability, macronutrient composition, body composition, supplement use, training volume, recovery practices, and other sport-specific behaviors can all contribute to variation in the gut microbiota. Nutritional strategies, including dietary modification and microbiota-targeted supplementation, are increasingly employed to support athlete health, immune function, and recovery. Because exercise, diet, and supplementation frequently co-occur in athletic populations, disentangling their independent contributions to gut microbiota composition remains challenging. Consequently, differences observed between athletic populations cannot readily be attributed to exercise alone and should be interpreted within the broader context of these interacting determinants. Evidence from studies examining exercise–microbiota interactions suggests that diet and probiotics may influence gastrointestinal and immune outcomes in athletes, although effects on gut microbiota composition are inconsistent and appear context dependent (42, 47). In trained populations, particularly, gut microbiota may exhibit relative stability, potentially limiting large-scale compositional changes following short-term interventions. This complexity highlights the importance of study designs that integrate dietary, physiological, and microbiome data to better characterize associations between specific training modalities and the gut microbiome (6).

Hybrid sports represent a distinct training model characterized by the simultaneous development of aerobic and resistance capacities. In the present study, “hybrid sport” refers operationally to a competition and training phenotype in which structured endurance running is combined with repeated functional resistance-based exercise stations within the same event format. Hyrox® was selected as a representative hybrid-sport model because it combines standardized 1-km running intervals with repeated functional strength and endurance tasks, providing a reproducible multimodal exercise stimulus. Although combat sports such as Muay Thai also require substantial aerobic and anaerobic capacity, they are primarily characterized by intermittent high-intensity striking exchanges, technical skill execution, contact stress, tactical decision-making, and weight-category practices, resulting in different physiological and training demands. Thus, while both Hyrox® and Muay Thai involve mixed aerobic and anaerobic metabolism, they represent distinct multimodal exercise contexts.

The physiological and neuromuscular adaptations associated with concurrent training have been extensively described, demonstrating that combined endurance and strength stimuli impose unique metabolic and recovery demands compared with single—modality training (46, 7). While these studies focus on host adaptations rather than the gut microbiota, they provide a rationale for considering hybrid athletes as a distinct population when investigating lifestyle—microbiota interactions.

Most studies investigating exercise and the gut microbiota have relied primarily on taxonomic profiling, to identify differences in microbial composition between athletes and sedentary individuals. However, in trained populations, where overall microbial diversity often remains relatively stable despite ongoing physiological adaptation, biologically relevant responses may occur through changes in microbial functional capacity rather than large shifts in community composition. Consequently, taxonomic profiling alone may not fully capture exercise-associated adaptations. To address this gap, full-length 16S rRNA sequencing was used to provide species-level taxonomic resolution across the study cohort, while exploratory shotgun metagenomic sequencing was performed in a subset of paired samples to investigate whether subtle taxonomic differences were accompanied by changes in microbial metabolic pathways involved in host–microbe interactions. These complementary approaches therefore allowed evaluation of both microbial composition and exploratory functional pathway differences that cannot be inferred from taxonomy alone (8). Despite these advances, relatively few studies have combined species-level taxonomic profiling with shotgun metagenomics in athletic populations, and most available research has focused on endurance-based disciplines. Consequently, it remains unclear whether athletes participating in hybrid sports exhibit distinct taxonomic characteristics and whether these are accompanied by detectable functional pathway differences following short-term dietary or microbiota-targeted interventions (9). Addressing this question requires both species-level taxonomic characterization and exploratory functional profiling to determine whether potential differences extend beyond microbial composition alone.

The aim of this study was therefore to evaluate baseline gut microbiota composition in Hyrox® athletes, as a representative hybrid-sport model, compared with Muay Thai athletes and sedentary adults, and to assess whether short-term dietary or microbiota-targeted interventions were associated with changes in microbial composition within each group. Species-level gut microbiota profiles were characterized using full-length 16S rRNA sequencing across the study cohort, while exploratory shotgun metagenomic analyses were performed in a subset of paired samples to investigate whether observed taxonomic differences were accompanied by corresponding differences in microbial metabolic pathways.

## 2 Material and methods

### 2.1 Participants and data collection

#### 2.1.1 Participant information

The study cohort comprised individuals engaged in different sport disciplines and training modalities, alongside non-athletic controls. Because the study groups differed with respect to training modality, dietary intervention, and microbiota-targeted supplementation, comparisons between groups should be interpreted as observational rather than causal. The cohort included ten adult Hyrox® athletes who underwent a three-month personalized dietary intervention designed to optimize nutritional intake and support gut microbiota health. This intervention was complemented by microbiota-targeted supplementation, including prebiotic and/or probiotic products, administered according to the nutritional programme. Fifteen adult Muay Thai fighters also participated in a three-month personalized dietary intervention but did not receive microbiota-targeted supplementation. Thirteen age-matched sedentary adults served as observational controls and were instructed to maintain their habitual dietary and lifestyle practices throughout the study period without receiving dietary counselling or supplementation. Details of the dietary intervention, microbiota-targeted supplementation (including composition, dosage, duration, and compliance monitoring) are provided in Supplementary Table S1. Individual participant-level data, including demographic and anthropometric characteristics [sex, age, body weight, height, and body mass index (BMI)], physiological measurements, dietary intake, food diary records, and microbiota-targeted supplementation received during the intervention, are provided in Supplementary Table S2. Because the interventions differed between athletic groups, longitudinal analyses were performed within each group rather than comparing intervention efficacy across groups. Demographic and anthropometric variables were used to describe the study cohort and were considered as potential confounders in downstream microbiota analyses. Age and BMI were explicitly accounted for in the statistical analyses to mitigate potential confounding due to inter-individual variability.

#### 2.1.2 Physical performance and physiological measurements

Physical performance and physiological measurements were analyzed only for participants with complete baseline and follow-up assessments; participants without complete physiological data were excluded. For participants included in these analyses, the same Concept2 rowing ergometer, damper setting, VO₂max estimation method, lactate analyzer, and testing protocol were used at both timepoints. Performance outcomes included 2000 m rowing ergometer performance at damper setting 5, recorded as completion time (seconds). The 2000m rowing ergometer test is a widely used and validated measure of whole-body endurance performance and has been extensively employed to investigate the physiological determinants of rowing performance in both elite and recreational athletes (10, 11).

Aerobic capacity of each participant was additionally estimated as maximal oxygen uptake (VO₂max) using a predictive formula provided by Concept2 (12):

$${\text{estimated \textbackslash{};VO}}_{2}\;\text{max}(\text{ml}\cdot {\text{kg}}^{-1}\cdot {\text{min}}^{-1})=\frac{Y\times 1000}{\text{Weight\textbackslash{}; (kg)}}$$

where Y depends on sex, body weight, training level and the 2000 m time trial performance. The equations used to calculate *Y* are presented in Supplementary Table S3. This approach is supported by Hagerman et al., who demonstrated a clear relationship between 2,000m rowing performance and VO₂max (13). Formula-derived values were retained as calculated to preserve consistency when computing ΔVO₂max.

Metabolic response to exercise was further characterized by post-exercise blood lactate concentration, which was measured 10 min after exercise. Blood lactate is a well-established marker of anaerobic contribution and metabolic stress during exercise, and standardized methodological frameworks exist for its measurement and interpretation in sport and exercise physiology (14, 15). To measure blood lactate concentrations, the Accutrend® Plus system from Roche Diagnostics (Mannheim, Germany) was used using Accutrend 3012654171 BM-Lactate Test Strips.

For participants with complete physiological data, we computed an exploratory combined efficiency score integrating estimated VO₂max and blood lactate measurements. Lactate values were inverted so that higher normalized values reflected better physiological performance, while VO₂max values were retained in their original direction. Both measures were normalized to a 0–1 scale before calculating the composite score.Change in combined efficiency from before to after the intervention (ΔCombinedEff) was calculated for each participant. Participants with a positive ΔCombinedEff (>0) were classified as ‘Improved', whereas those with no change or a negative ΔCombinedEff (≤0) were classified as ‘Not improved’; this classification defines the Improvement Status. This exploratory classification was introduced to facilitate stratified analyses based on overall physiological response rather than individual physiological metrics. Accordingly, all analyses using the Improvement Status variable should be interpreted as hypothesis-generating.

#### 2.1.3 Dietary assessment

For the Hyrox® athlete group, detailed dietary intake data were collected throughout the three-month intervention period. Participants recorded their daily food and beverage intake, using dietary logs collected at the end of the three-month intervention. This was subsequently quantified to estimate total energy intake (kcal) and macronutrient composition: protein (g), carbohydrates (g), sugars (g), fiber (g), and fat (g). Dietary intake data were used to characterize individual dietary patterns over the intervention period and to examine potential associations between nutrient intake and gut microbiota composition and functional potential. The individual dietary intake data used for these analyses, together with the corresponding food diary tables, are provided in the additional sheets of Supplementary Table S2.

#### 2.1.4 Ethics approval statement

The study was approved by the University of Malta, Msida, Malta, Faculty research ethics committee FHS-2023-00080 on the 30th October 2023. All participants were informed about the study and written and/or verbally informed consent was obtained. Personal data were protected in accordance with principles of confidentiality.

### 2.2 Sample collection and microbial DNA extraction

Fecal samples were collected from all participants at baseline, prior to the start of the study (*n* = 38). Follow-up fecal samples were collected after completion of the 3-month observation period from participants undergoing the dietary intervention, including available Hyrox® athletes (*n* = 9) and the available Muay Thai fighters (*n* = 6). In the sedentary group, follow-up sampling was not systematically planned for all participants; two sedentary participants provided a follow-up sample to allow a descriptive assessment of temporal variation in the absence of intervention. Participants in the intervention groups who did not provide a follow-up fecal sample had either withdrawn from the study or decided not to continue the intervention. All samples were obtained with informed consent from the participants and were fully anonymized. Details of participant inclusion, follow-up sample availability, and reasons for missing follow-up samples are provided in Supplementary Table S4.

Stool samples were collected using the Danastool Sample Collection Microbiome Kit (Danagen, Barcelona, Spain). For each sample, a 200 µL aliquot was used for DNA extraction. Total DNA was extracted using the MagMax™ Microbiome Ultra Nucleic Acid Isolation Kit (Applied Biosystems, Carlsbad, CA, USA) in combination with the KingFisher™ Flex Purification System (Thermo Fisher Scientific, Waltham, MA, USA), following the manufacturer's protocol. Mechanical lysis was performed by bead-beating at 6 m/s for 40 s for two cycles using the MP FastPrep-24™ 5G homogenizer (MP Biomedicals, Irvine, CA, USA). DNA was eluted in 100 µL of elution buffer. DNA concentration was determined using a Qubit™ 4 Fluorometer (Thermo Fisher Scientific) with the 1× dsDNA High Sensitivity Assay Kit.

Extracted DNA was used for downstream 16S rRNA gene sequencing to profile gut microbiota composition. Shotgun metagenomic sequencing was performed only on participants with complete paired baseline and follow-up fecal samples available for metagenomic analysis (*n* = 15; see Supplementary Table S1) to characterize functional potential, including genes and metabolic pathways.

### 2.3 Libraries preparation

Full-length 16S rRNA gene sequencing and library preparation were performed following the protocol previously described (16). Briefly, full-length 16S rRNA genes (∼1,500 bp) were amplified, barcoded, and sequenced using Oxford Nanopore Technologies (ONT) long-read sequencing chemistry. PCR amplification, bead-based purification, barcoding, and adapter ligation were carried out following the validated workflow described in the reference study (16), which incorporates LongAmp® Taq polymerase (New England Biolabs), AMPure XP bead clean-up steps (Beckman Coulter), a custom set of phosphorylated barcoding primers allowing sample multiplexing, and ligation-based library construction using the SQK-LSK114 kit (ONT). Sequencing was performed on R10.4.1 flow cells using a GridION Mk1B platform with real-time super-accurate basecalling and live demultiplexing enabled. This standardized workflow has been previously optimized and validated for full-length 16S rRNA profiling in fecal microbiota samples, ensuring consistency and comparability across studies.

Whole-genome (shotgun) metagenomic library preparation and sequencing were performed following the PISTE^TM^ workflow previously described (17). Briefly, microbial DNA was enzymatically fragmented via transposase-mediated tagmentation, barcoded, and PCR-amplified using Rapid PCR barcoding kit SQK-RPB114.24 (Oxford Nanopore Technologies). Libraries were purified using AMPure XP beads, pooled in equimolar amounts, and sequencing adapters were ligated according to the manufacturer's instructions. Sequencing was performed on R10.4.1 flow cells using a GridION Mk1B platform (Oxford Nanopore Technologies), enabling long-read whole-genome metagenomic profiling. This protocol has been previously optimized and validated for low-biomass microbiome samples (17).

### 2.4 Data analysis workflow

For 16S rRNA gene sequencing, native pod5 files generated by Oxford Nanopore sequencing were live basecalled and demultiplexed using Guppy v6.1.5 (Oxford Nanopore Technologies) in super-accurate mode, with a minimum quality threshold of 12. Sequencing read quality and length distributions were evaluated using NanoPlot v1.44.1 (18). The resulting fastQ reads were then processed through a custom bioinformatics workflow, adapted from our previous publication (16). Adapters and primer sequences were removed using cutadapt v5.0 (19), followed by filtering based on read length and quality with the DADA2 R package v1.22.0 (20). Only reads between 1,300 and 1,600 nucleotides and with a maximum expected error (maxEE) of 25 were retained. Potential chimeras were detected and eliminated using a *de novo* strategy with minimap2 v2.28 (-x ava-ont, max seed distance 500) (21) in combination with yacrd v1.0.0 (default settings) (22). Species-level taxonomy was assigned using emu v3.5.2 (23) against its internal emudb_345.v2 database. The final tab-separated output was imported into R for downstream statistical analyses.

Raw metagenomic sequences were initially examined to remove potential human DNA contamination. FastQ reads were aligned against the telomere-to-telomere human reference genome T2T-CHM13 v2.0 (24) using minimap2 (v2.28-r1209) with the -ax map-ont preset and -f 1,000 parameter settings (21). Reads mapping to the human genome were subsequently discarded using samtools (v1.21; samtools view -b -f 4) (25), ensuring that only non-human sequences were retained for downstream analysis. The filtered microbial reads were taxonomically assigned with Kraken2 (v2.1.3) (26) using the core_nt database. This database comprises an extensive collection of nucleotide sequences from NCBI RefSeq, GenBank, TPA, and PDB, encompassing archaeal, bacterial, viral, fungal, protozoan, and human entries (27). For functional profiling, gene prediction was carried out in metagenomic mode using Prodigal (v2.6.3) (28) on the host-depleted reads. Functional annotation and KEGG Orthology (KO) assignment were performed with eggNOG-mapper (v2.1.13) (29) based on the eggNOG database (v5.0.2), while sequence similarity searches were conducted using DIAMOND (v2.1.13) (30). DIAMOND results were filtered with stringent criteria: E-value ≤ 1 × 10^−30^, minimum query and subject coverage of 25%, and sequence identity ≥50%. The curated KO annotations were then restricted to pathways of interest and processed with MinPath (v1.6) (31) to infer and report metabolic pathway reconstructions.

### 2.5 Statistical analysis

All statistical analyses were performed in R (v. 4.5.0) (32) within the RStudio (v. 421) (33) environment. Differences in demographic and clinical variables between groups were evaluated using Chi-square tests for categorical variables (sex) and Kruskal–Wallis tests for continuous variables (age; BMI). A *p*-value < 0.05 was considered statistically significant.

For diversity analyses, the full-length 16s dataset was rarefied to a uniform sequencing depth of 16,000 reads per sample using the rarefy even depth function from phyloseq (v. 1.52.3) (34)*.* To reduce noise from low-abundance taxa, only taxa with at least 5 counts across samples were retained using the filter taxa function from phyloseq. Alpha diversity was calculated using the Shannon and Simpson indices, along with observed richness (number of unique taxa), all computed with phyloseq. Differences between groups were assessed using Kruskal–Wallis tests from the stats package (v. 4.5.0). Beta diversity analyses were performed on Hellinger-transformed counts (using microbiome v. 1.30) (35) and visualized using Principal Coordinates Analysis (PCoA) based on Bray-Curtis dissimilarities. Ordination plots were generated with microbiome and ggplot2 (v. 3.5.2) (35, 36). Community-level differences between groups were tested using PERMANOVA with the adonis2 function (vegan v. 2.6.10), and group dispersion was assessed using betadisper followed by permutest in vegan to evaluate homogeneity of variances (37).

Differential abundance at the species-, family- and phylum-level was analyzed using DESeq2 (v. 1.48.2) (38). Taxa were aggregated at each taxonomic level using the tax glom function from the phyloseq package (34).

For cross-sectional comparisons at baseline (Before), the raw taxonomic abundance table (taxon-level counts) were converted to DESeq2 format, with Sport category as the main variable and sex as a covariate. Library size was normalized using the median-of-ratios method, and models were fitted with the Wald test using parametric dispersion estimates. Pairwise contrasts between sport categories were extracted, and *p*-values were adjusted with the Benjamini-Hochberg procedure.

For paired analyses (Before vs. After), differential abundance was assessed separately within each subgroup. The dataset was split by Sport category (Hyrox® and Fighters) and by Improvement status (Yes and No). DESeq2 models were fitted for each subset using participant identity as a blocking factor and Timepoint as the variable of interest (design=Participant ID + Timepoint). Size factors, dispersion modeling, and Wald tests were conducted as described above. Benjamini–Hochberg correction was applied to account for multiple testing, and taxa with non-missing adjusted *p*-values were retained for downstream analyses.

For visualization with ggplot2 (36), relative abundances were expressed as percentages, and group means ± 95% confidence intervals (95% CI) were reported for each timepoint.

Shotgun metagenomic analyses were performed on paired fecal samples to examine changes in microbial genes and functional pathways before and after the intervention. Protein-level hits from EggNOG-based minpath outputs were extracted and converted into relative abundances for each sample (proportion of hits out of total hits). Paired comparisons between Before and After within each sport category were performed using Wilcoxon tests from the stats package (v. 4.5.0). All *p*-values were adjusted using the Benjamini–Hochberg method. Protein relative abundances were used for visualization with ggplot2 (36).

For functional pathways, presence or absence was recorded for each participant, and pathway prevalence was calculated as the proportion of participants in which the pathway was detected. Differences between Before and After were expressed as absolute changes in prevalence. McNemar's tests from the stats package (v. 4.5.0) were used to assess pathway-level shifts separately for the Hyrox® and Fighter subgroups, with Benjamini–Hochberg correction applied for multiple comparisons. Pathway prevalence was used for visualization with ggplot2 (36).

For exploratory analyses of associations between nutrient intake, supplementation exposure, and paired taxonomic changes, paired changes in taxon abundances (ΔTaxa) were calculated for each participant by subtracting relative abundance at the Before timepoint from that at the After timepoint. Analyses were performed at the species, family, and phylum levels, as these taxonomic ranks provide a balance between resolution and biological interpretability in 16S rRNA gene studies. Taxa were aggregated at these levels, filtered to retain those with a mean relative abundance ≥ 0.1%, and converted to relative abundances as the sole normalization step. A log1p transformation was subsequently applied for variance stabilization prior to downstream analyses. The resulting tables were merged with participant metadata using functions from the tidyverse package (v. 2.0.0) (39).

*P*-values were adjusted for multiple testing using the Benjamini–Hochberg procedure across all nutrient–taxon association tests. Because no associations remained significant after false-discovery-rate correction, exploratory correlations with an absolute effect size (|*ρ*| > 0.5) and nominal *p* < 0.05 are presented for descriptive purposes. Effects of prebiotic supplementation (GOS and FOS; binary variables) on ΔTaxa were evaluated using Wilcoxon rank-sum tests, reporting median differences between supplemented and non-supplemented individuals as effect sizes. Benjamini–Hochberg correction was applied separately to the predefined nutrient–taxon (258 Spearman correlation tests across 43 taxa and 6 dietary variables) and supplementation–taxon (86 Wilcoxon tests across 43 taxa and 2 supplementation variables) testing families. Because no supplementation-associated taxa remained significant after multiple-testing correction, exploratory results with a nominal *p* < 0.05 and median difference >0.5 are presented for descriptive purposes. Probiotic supplementation effects were not evaluated due to an insufficient number of participants using them.

Exploratorydiet- and supplement-associated ΔTaxa were visualized as heatmaps using ggplot2 (36). This multi-level approach allowed visualization of exploratory associations between dietary intake, prebiotic supplementation, and paired changes in gut microbial taxa in Hyrox® athletes.

## 3 Results

### 3.1 Study population

**Figure 1:**
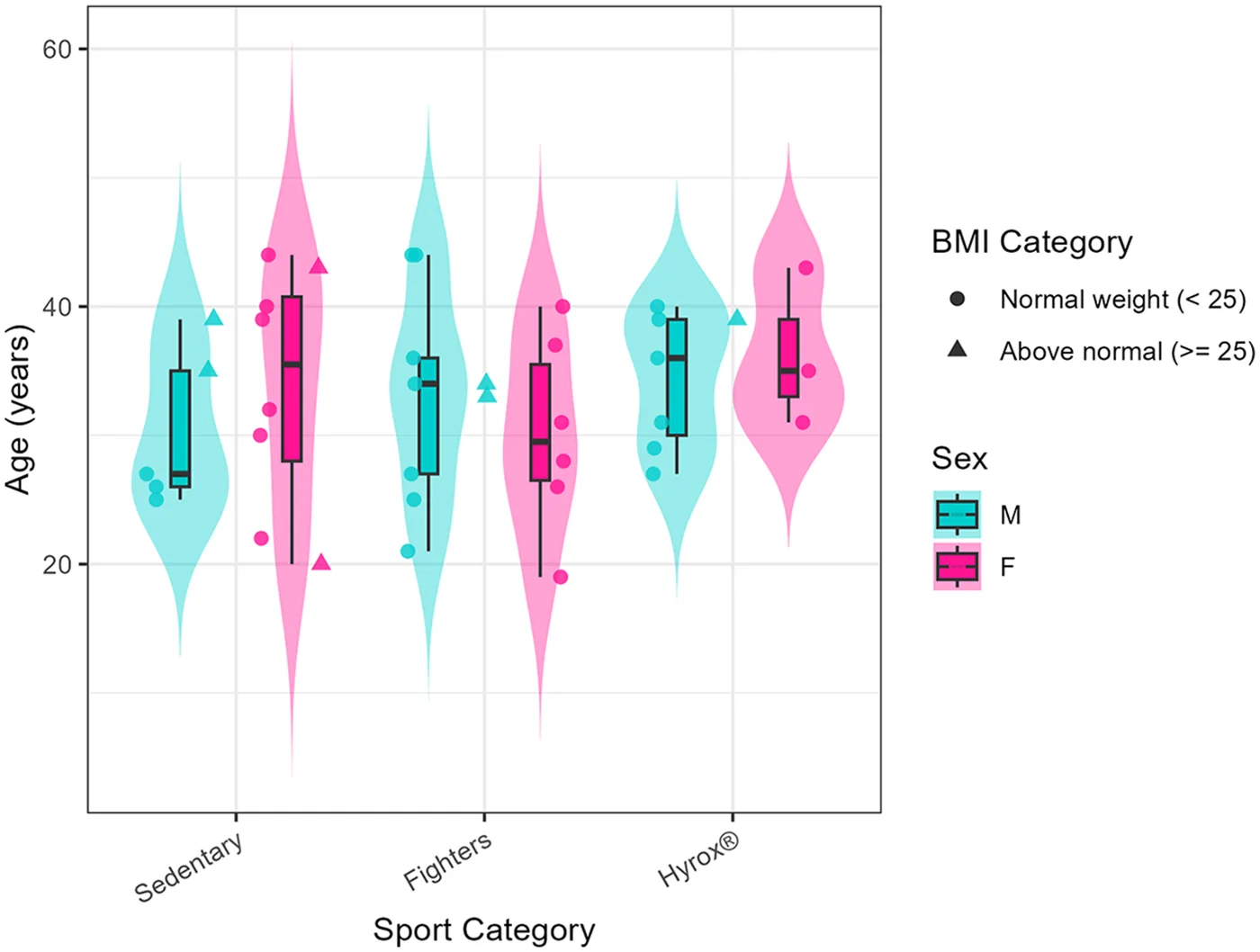
Demographic distribution of participants across sport groups. Violin plots illustrate the age distribution within sedentary, fighters and Hyrox® participants. Data are stratified by sex, with male participants represented in blue and female participants in pink. Individual body mass index (BMI) categories are represented by circles for BMI < 25 and triangles for BMI ≥ 25.

A total of 38 individuals were recruited, spanning three physical activity profiles: 13 sedentary adults, 15 fighters, and 10 Hyrox® athletes. Participants covered a broad adult age range, with fighters being the youngest group (31.9 ± 7.6 years), followed closely by sedentary individuals (32.5 ± 8.1 years), while Hyrox® athletes were the oldest (35.0 ± 5.3 years). Sex distribution differed between groups: fighters showed a male predominance (9/15 males), as did Hyrox® athletes (7/10 males), whereas the sedentary group was female-dominant (8/13 females). BMI values were within the normal weight range, as defined by the World Health Organization (18.5–24.9 kg/m^2^) (40), and were remarkably similar across sport categories, indicating that putative differences in gut microbiota are unlikely to be driven by body composition alone (Table 1, Figure 1).

**Table 1:** Demographic and clinical characteristics of study participants.

Variable	Sedentary (*n* = 13)	Muay Thai fighters (*n* = 15)	Hyrox® (*n* = 10)
Sex (M/F)	5/8	9/6	7/3
Age (mean ± SD)	32.5 ± 8.1	31.9 ± 7.6	35.0 ± 5.3
BMI (mean ± SD)	22.5 ± 4.0	23.1 ± 2.3	23.3 ± 1.8

Summary of sex, age and BMI across groups. Data are shown as mean ± SD or *n* (number of particapants).

To evaluate potential demographic confounders (which are not required for paired analyses, as participants serve as their own controls), age, sex, and BMI were compared across the Sedentary, Fighters, and Hyrox® groups. No significant differences were observed for age (Kruskal–Wallis *χ*^2^ = 1.05, *p* = 0.595) or BMI (Kruskal–Wallis *χ*^2^ = 1.55, *p* = 0.461), and sex distribution was also comparable between groups (*χ*^2^ = 2.50, *p* = 0.287). However, the absence of statistically significant differences does not exclude the possibility of residual confounding, particularly given the unequal sex distribution across groups. Accordingly, age, sex, and BMI were retained as covariates in all beta diversity models to account for their potential influence on microbial community composition. Because these demographic variables were not associated with alpha diversity, they were not included as covariates in the alpha diversity analyses.

### 3.2 Sequence data quality and filtering

#### 3.2.1 Full-length 16S rRNA amplicon sequencing dataset

The raw full-length 16S rRNA amplicon sequencing dataset comprised over 7.7 million reads, with an average read length of 1,372 bp (N50 = 1,491 bp) and a mean Q-score of 19.2. Reads failing quality criteria (such as chimeric sequences or those outside the 1,300–1,600 bp length range) were excluded. Following this filtering, approximately 4.2 million high-quality reads remained, distributed across 55 samples, with an average of 73,306 reads per sample (range: 16,625–322,648). These retained reads had an average length of 1,448 bp (N50 = 1,449 bp) and an average Q-score of 21.5.

Taxonomic assignment was then performed using emu. To standardize sequencing depth for downstream diversity analyses, rarefaction was applied to 16,000 reads per sample, based on the minimum sequencing depth observed (16,625 reads), retaining 21.9% of the high-quality reads. Only taxa with at least five reads were included. This rarefaction slightly reduced dataset sparsity from 0.76 to 0.71, while maintaining approximately 79.9% of species, indicating a minor loss of low-abundance taxa.

#### 3.2.2 Whole genome sequencing dataset

The shotgun metagenomic dataset comprised a total of 6,359,526 high-quality reads across 30 samples. The number of reads per sample ranged from 14,244 to 986,671, with a mean of 239,627 reads per sample. The retained sequences had an average length of 3,579 bp (N50 = 4,008 bp) and a mean quality score of 18.6, reflecting high-quality long-read data suitable for downstream metagenomic analyses.

### 3.3 Diversity metrics and differential abundance analysis of baseline microbiota

Before evaluating the effects of the interventions, the baseline gut microbiota of the participants was first compared in order to determine whether the groups differed in their microbial composition prior to any intervention.

**Figure 2:**
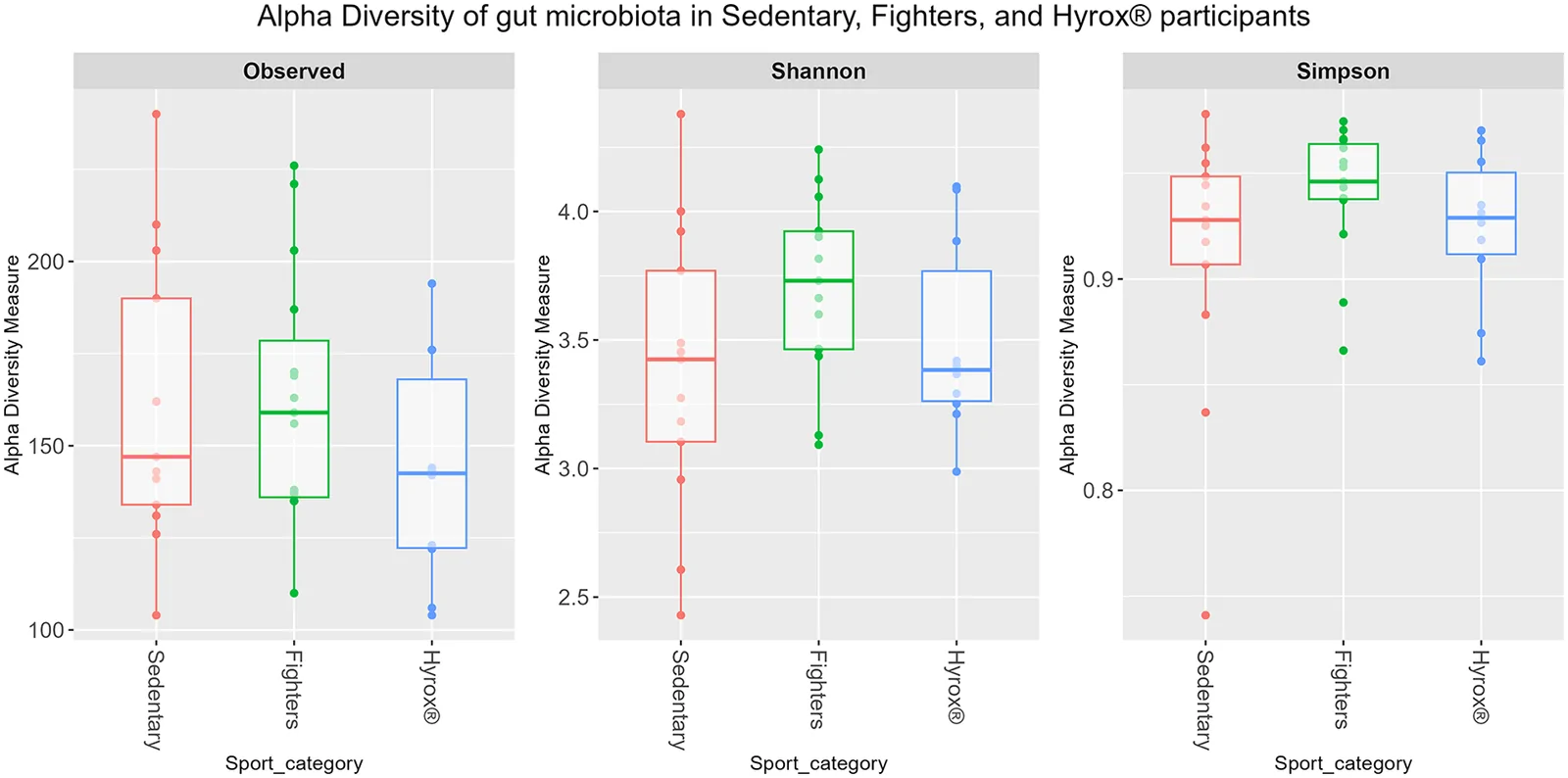
Boxplots of species-level alpha diversity (observed richness, shannon, and simpson indexes) across sport categories: sedentary (red), fighters (green), and hyrox® (blue).

Baseline alpha diversity was assessed using rarefied 16S data for Observed richness, Shannon, and Simpson indices (Figure 2). Statistical testing was performed using non-parametric methods (Kruskal–Wallis with Dunn post-hoc). No significant differences in baseline alpha diversity were detected for Observed richness (*χ*^2^ = 1.68, *p* = 0.432), Shannon diversity (*χ*^2^ = 3.51, *p* = 0.173), or Simpson evenness (*χ*^2^ = 3.37, *p* = 0.185). Estimated marginal means obtained from generalized linear models were consistent with these findings. For Observed richness, adjusted means (95% CI) were 162 (143–182) for Sedentary participants, 164 (146–182) for Fighters, and 139 (117–162) for Hyrox® athletes. For Shannon diversity, adjusted means were 3.40 (3.16–3.64), 3.72 (3.49–3.94), and 3.46 (3.18–3.73), respectively. Corresponding Simpson indices were 0.913 (0.888–0.939), 0.943 (0.919–0.967), and 0.921 (0.892–0.951). Dunn *post-hoc* tests with Benjamini–Hochberg adjustment confirmed the absence of pairwise differences. These results indicate that baseline species richness, diversity, and evenness were similar across Sedentary, Fighters, and Hyrox® participants.

**Figure 3:**
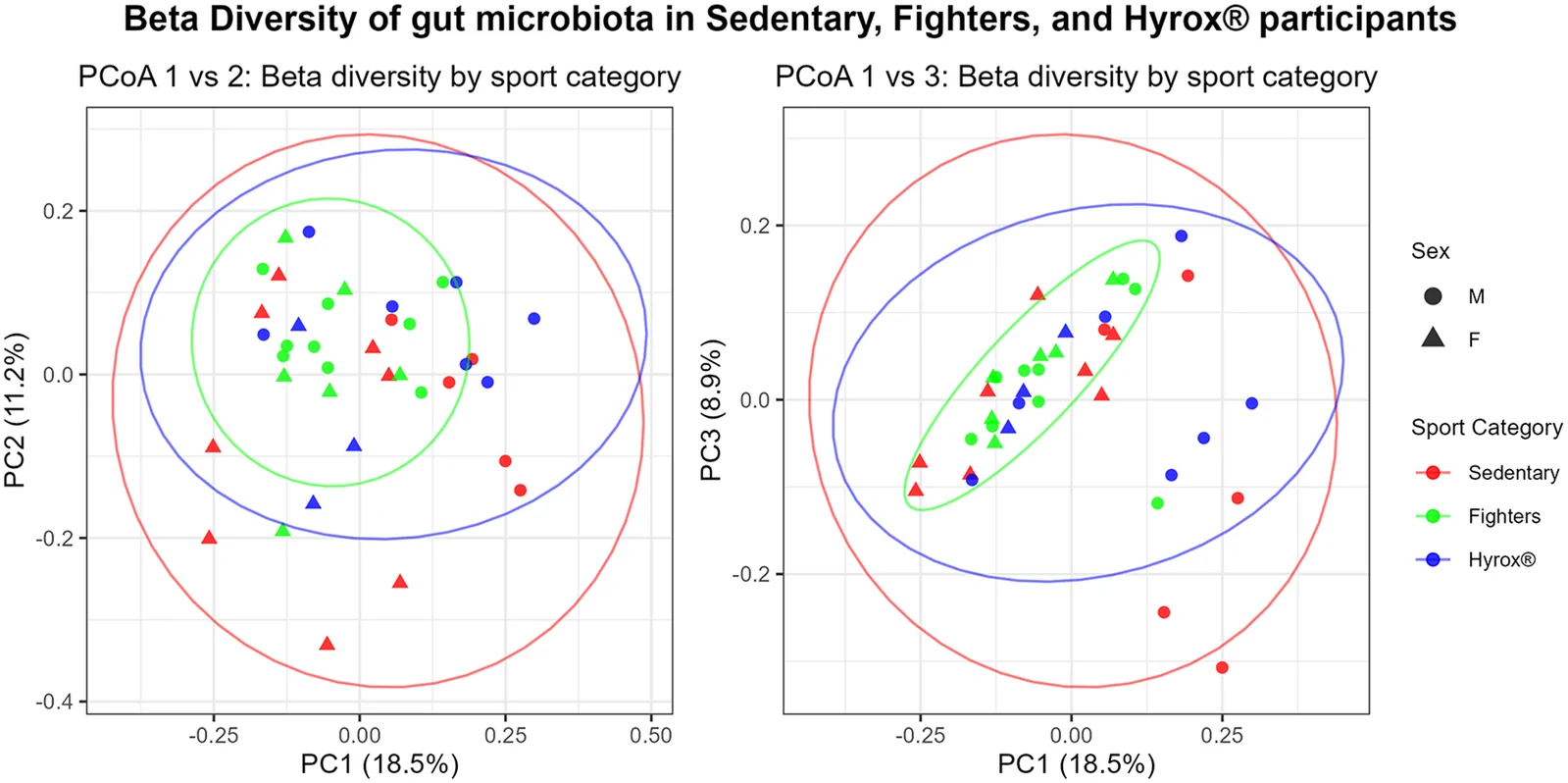
Principal coordinate analysis (PCoA) plots show beta diversity (bray–curtis distance) computed on rarefied and hellinger-transformed data at the species level, with ellipses representing 95% confidence intervals for each sport category. Samples are colored according to sport category: Sedentary (red), Fighters (green), and Hyrox® (blue). Points are shaped according to sex (circles: males, triangles: females).

Beta diversity was assessed using Bray–Curtis dissimilarities on rarefied and Hellinger-transformed data and visualized by PCoA (Figure 3). Initial PERMANOVA analyses using single explanatory variables showed that sex was the strongest determinant of microbial community composition (*R*^2^ = 0.069, *p* = 0.003). Sport category displayed a trend toward significance (*R*^2^ = 0.070, *p* = 0.096), while age (*R*^2^ = 0.038, *p* = 0.102) and BMI (*R*^2^ = 0.035, *p* = 0.139) were not significantly associated with overall community structure. Because age and BMI showed no significant effects and did not differ across sport categories (see part 3.1.), they were excluded from the multifactorial model. In the multifactorial model including sport category and sex, sex remained highly significant (*R*^2^ = 0.068, *p* = 0.001), while sport category showed a marginal effect (*R*^2^ = 0.070, *p* = 0.060). Group dispersion was assessed to ensure homogeneity of variance. Sedentary participants showed significantly greater gut microbiota dissimilarity compared with Fighters (*p* = 0.003), while Hyrox® participants differed significantly from Fighters (*p* = 0.032) but not from Sedentary individuals (*p* = 0.284). Sex groups showed homogeneous dispersion (*p* = 0.649). These results indicate that the spread of microbiota composition differs across sport categories and should be considered when interpreting PERMANOVA results. As a sensitivity analysis, PERMANOVA was repeated separately in male and female participants. Among males, sport category was significantly associated with gut microbiota composition (PERMANOVA: *F* = 1.88, *R*^2^ = 0.173, *p* = 0.010). In contrast, no significant association was detected among females (PERMANOVA: *F* = 0.81, *R*^2^ = 0.104, *p* = 0.791). Given the limited number of female Hyrox® participants (*n* = 3), the female-specific analysis should be interpreted cautiously and considered exploratory. Overall, these findings suggest that sex contributes strongly to gut microbiota compositional differences, sport category has a marginal effect, and differences in dispersion across sport categories (particularly the higher variability in Sedentary participants) should be noted.

Differential abundance analysis was performed using DESeq2, with sport category included as the main variable and sex included as a covariate to account for its significant influence on microbial community composition. This approach allowed identification of taxa associated with sport category independently of sex effects.

**Figure 4:**
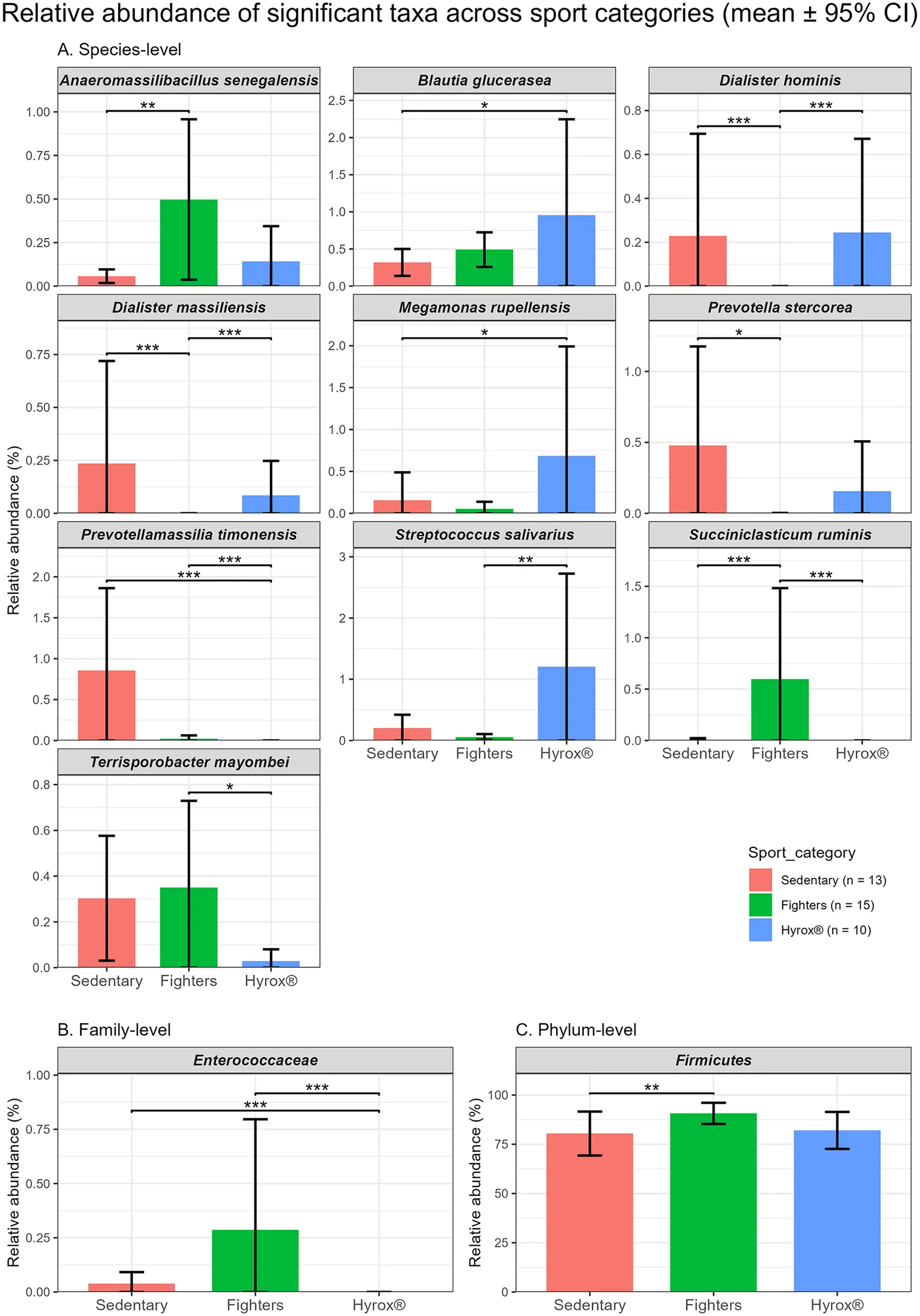
Relative abundance of significant gut microbiota taxa across sport categories. Bars represent the mean relative abundance for each species **(A)**, family **(B)**, and phylum **(C)**, with error bars showing ± 95% confidence intervals. Asterisks indicate significant differences in abundance between groups (Fighters, Hyrox®, Sedentary) based on pairwise DESeq2 Wald tests using adjusted *p*-values, with *** *p*.adj < 0.001, ** *p*.adj < 0.01 and * *p*.adj < 0.05.

Analysis of the gut microbiota revealed distinct taxa associated with different sport categories, with several species repeatedly observed across multiple groups, indicating patterns of differential abundance observed across comparisons (Figure 4A). Only taxa with mean relative abundance >0.1% across samples were considered. In Fighters vs. Hyrox®, taxa reduced in Fighters (i.e., higher in Hyrox®) included *Dialister hominis*, *Dialister massiliensis*, and *Streptococcus salivarius*, whereas *Succiniclasticum ruminis*, *Prevotellamassilia timonensis*, and *Terrisporobacter mayombei* were reduced in Hyrox® (i.e., higher in Fighters). Log₂ fold changes were large (±4–27), highlighting pronounced differences in abundance. Comparisons involving Sedentary participants showed reduced *Succiniclasticum ruminis*, *Anaeromassilibacillus senegalensis*, and *Blautia glucerasea* in Sedentary individuals (i.e., higher in active participants), whereas *Dialister hominis*, *Dialister massiliensis*, and *Prevotella stercorea* were reduced in active participants (i.e., higher in Sedentary individuals). Some taxa, such as *Prevotellamassilia timonensis*, were reduced in Hyrox® relative to Sedentary individuals (i.e., higher in Sedentary participants).

Notably, certain taxa appeared consistently across multiple comparisons, suggesting broader associations with physical activity rather than strict sport-specifici associations. For example, both *Dialister hominis* and *Dialister massiliensis* were consistently reduced in Fighters compared to both Hyrox® and Sedentary groups, indicating higher abundance in the latter groups and suggesting they are not specific markers of combat-based training. Conversely, *Prevotellamassilia timonensis* and *Succiniclasticum ruminis* were consistently reduced in Hyrox® participants relative to other groups, indicating higher abundance in Fighters or Sedentary individuals rather than Hyrox®. Sedentary individuals showed higher abundance of taxa such as *Dialister hominis*, *Dialister massiliensis*, and *Prevotella stercorea*, whereas taxa such as *Anaeromassilibacillus senegalensis* and *Blautia glucerasea* were relatively reduced in this group compared to active participants. Median relative abundances, interquartile ranges, prevalence, and sample sizes for all candidate taxa are provided in Supplementary Table S5. Several candidate taxa exhibited low prevalence and median relative abundances of zero across one or more groups, a pattern consistent with the sparse nature of microbiome compositional data; significant differences in these taxa likely reflect abundance shifts in a subset of participants rather than uniform changes across the entire cohort. Therefore, these findings should be interpreted as exploratory and considered candidate taxa requiring validation in larger, independent studies rather than biomarkers of sport participation.

Analysis at the family level identified Enterococcaceae as the only family showing significant differential abundance between sport categories (Figure 4B). Enterococcaceae was not detected in any Hyrox® participant (prevalence: 0%), whereas it was detected in 26.7% of Fighters and 38.5% of Sedentary participants (Supplementary Table S5). Consequently, DESeq2 estimated very large negative log₂ fold changes for Hyrox® compared with Fighters (−23.7) and Sedentary participants (−26.9) (adjusted *p* < 0.001 for both comparisons). Median relative abundance was 0% in all groups, indicating that these differences were driven by the absence of Enterococcaceae in the Hyrox® group and its detection in a subset of Fighters and Sedentary participants rather than by widespread differences across the entire cohort. Accordingly, Enterococcaceae should be regarded as a candidate family identified in this exploratory analysis that requires validation in larger, independent cohorts.At the phylum level (Figure 4C), *Firmicutes* showed a modest but statistically significant increase in Fighters compared to Sedentary participants (log₂ fold change = 0.74, adjusted *p* = 0.008) (Supplementary Table S5). However, the relatively small effect size compared to those observed at lower taxonomic levels suggests that microbiota differences between sport categories are more pronounced at finer taxonomic resolution, particularly at the family and species levels.

### 3.4 Paired analyses before and after the intervention

**Figure 5:**
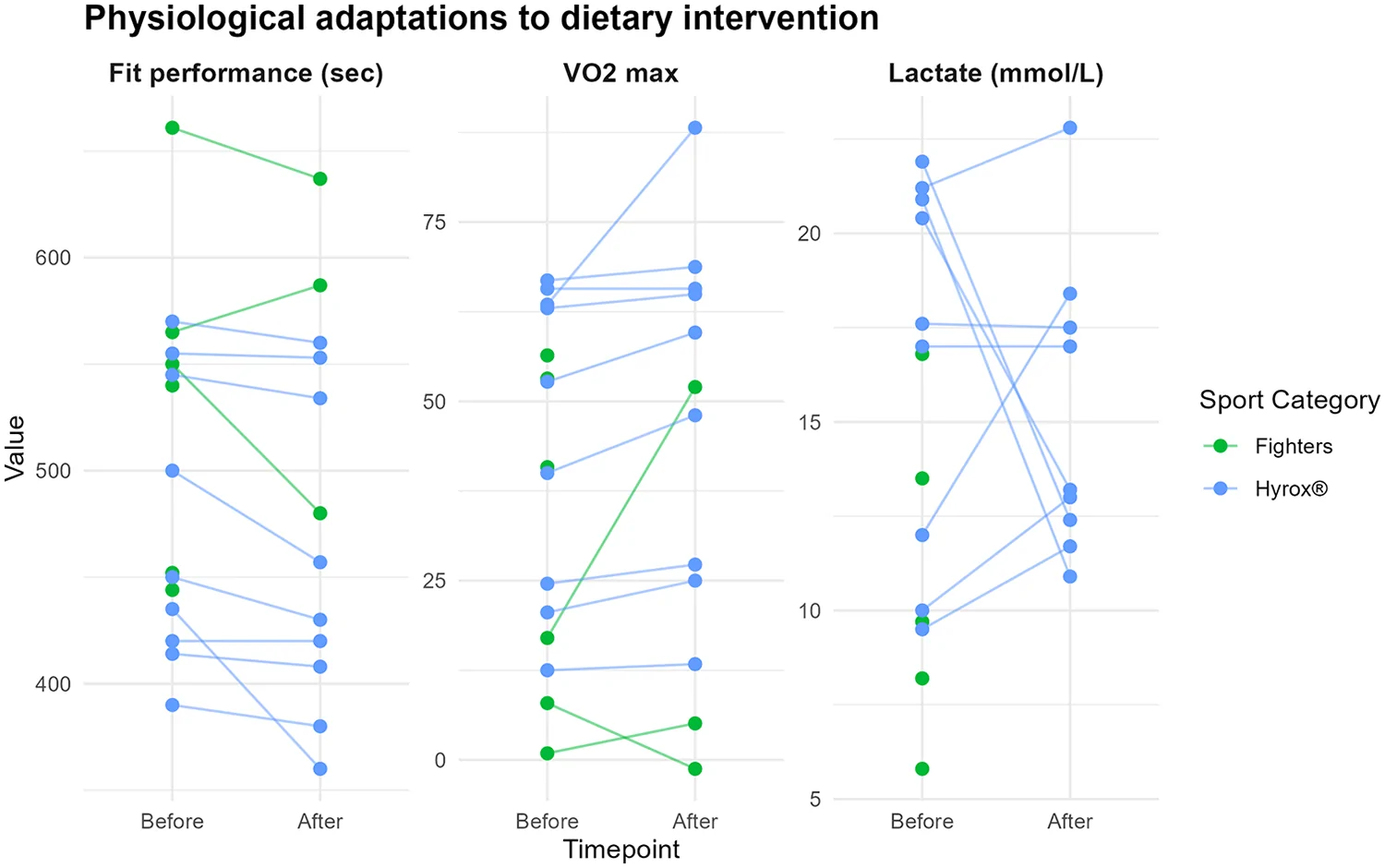
Physiological measurements before and after the study period. Individual participant trajectories are shown for fit performance (seconds), VO₂ max (), and blood lactate (mmol/L) at each timepoint, colored by sport category. Points represent raw values. Lines connect repeated measurements within participants, highlighting individual responses.

**Figure 6:**
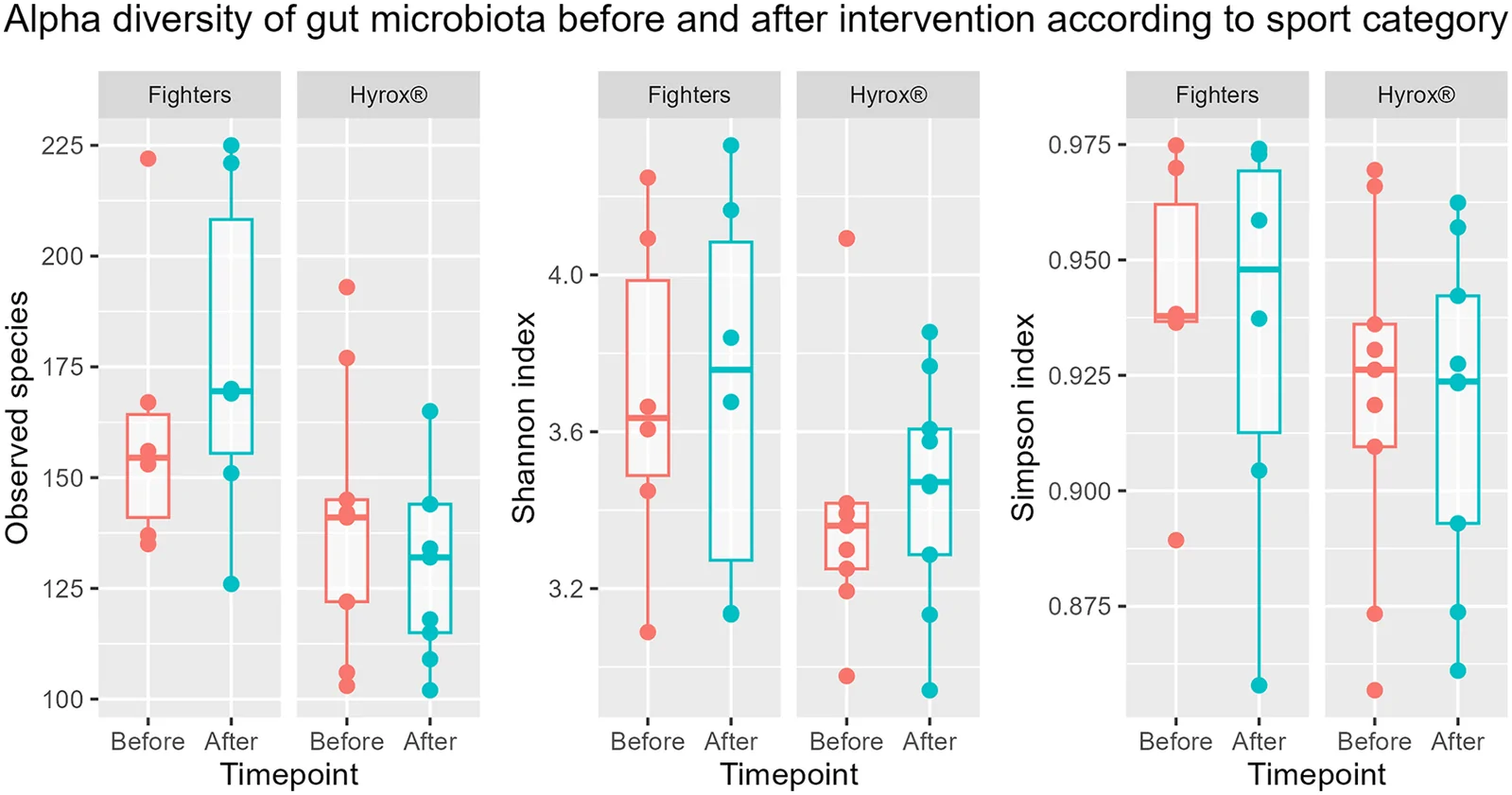
Alpha diversity of the gut microbiota before and after intervention across sport categories. Observed richness, Shannon and Simpson indices are shown for each participant group. No significant changes were observed over time or between sport categories.

We assessed changes in key performance and metabolic measures before and after the 3-month intervention in participants with paired measurements. Across all paired participants (*n* = 12 for rowing performance and estimated VO₂max; *n* = 9 for lactate), rowing performance improved by a mean of 20.8 s (95% CI: 2.6–39.0 s; *p* = 0.023), and estimated VO₂max increased by 6.78 mL·kg⁻^1^·min⁻^1^ (95% CI: −0.71–14.27 mL·kg⁻^1^·min⁻^1^; *p* = 0.037). Blood lactate showed no significant overall change (mean *Δ* = −1.51 mmol/L, 95% CI: −6.05–3.03 mmol/L; *p* = 0.624).When analysed by sport category, paired data were available for three Fighters and nine Hyrox® athletes for rowing performance and estimated VO₂max, whereas lactate measurements were available only for Hyrox® athletes (*n* = 9). Hyrox® athletes showed significant improvements in rowing performance (mean Δ = −19.7 s, 95% CI: −38.4 to −0.9 s; *p* = 0.014) and estimated VO₂max (mean Δ = 5.71 mL·kg⁻^1^·min⁻^1^, 95% CI: −0.13–11.55 mL·kg⁻^1^·min⁻^1^; *p* = 0.014), while lactate concentrations did not change significantly (mean Δ = −1.51 mmol/L, 95% CI: −6.05–3.03 mmol/L; *p* = 0.624). In Fighters, no statistically significant within-group changes were detected for rowing performance (mean Δ = −24.0 s, 95% CI: −138.3–90.3 s; *p* = 0.50) or estimated VO₂max (mean *Δ* = 10.0 mL kg⁻^1^·min⁻^1^, 95% CI: −46.3–66.3 mL kg⁻^1^·min⁻^1^; *p* = 0.75), although these analyses should be interpreted cautiously because only three paired observations were available.To determine whether the response to the intervention differed between sport categories, a linear mixed-effects model including the Timepoint×Sport category interaction was fitted. No significant interaction was observed for rowing performance (*p* = 0.764) or estimated VO₂max (*p* = 0.401), indicating that the magnitude of change did not differ significantly between Fighters and Hyrox® athletes. Similarly, direct comparisons of change scores between groups were not significant for rowing performance (*p* = 0.853) or estimated VO₂max (*p* = 1.000). Based on the integrated combined efficiency score (*Δ*CombinedEff), eight participants were classified as “Improved” and four as “Not improved” (Table 2). Individual trajectories are presented in Figure 5. We evaluated the longitudinal gut microbiota alpha diversity shifts using Observed richness, Shannon, and Simpson indices (Figure 6). Linear mixed-effects models were employed to account for repeated measures within participants, with fixed effects including Timepoint (Before vs. After) and Sport category. Across the cohort, none of the alpha diversity metrics changed significantly over time. Observed richness did not vary between timepoints (Estimate = 0.27, *F* = 0.001, *p* = 0.981), nor did Shannon (Estimate = 0.010, *F* = 0.009, *p* = 0.928) or Simpson indices (Estimate = −0.004, *F* = 0.139, *p* = 0.715). Sport category was associated with a significant effect on Observed richness (Hyrox® vs. Sedentary: Estimate = −35.22, F = 9.48, *p* = 0.0088), while Shannon (Estimate = −0.249, *F* = 2.12, *p* = 0.169) and Simpson (Estimate = −0.018, *F* = 1.35, *p* = 0.266) indices were not significantly influenced. There were no significant interactions with Timepoint for any metric. Overall, these findings indicate that no statistically significant changes in overall microbial richness or diversity were detected over the intervention period. While Hyrox® participants showed lower Observed richness than Sedentary participants, Shannon and Simpson diversity were not significantly associated with sport category or timepoint. These findings suggest that any effects of the intervention or sport category on alpha diversity were limited or too small to be detected in the present cohort.

**Table 2:** Combined efficiency score for each participant with complete physiological data.

Participant_code	Before	After	Delta_CombinedEff	Improvement
G_016	0.0242	0.0709	0.0468	Yes
G_021	0.551	0.000	−0.551	No
G_023	0.279	0.596	0.317	Yes
G_034	0.527	0.530	0.00294	Yes
G_035	0.753	0.826	0.0733	Yes
G_036	0.148	0.453	0.304	Yes
G_037	0.437	0.741	0.305	Yes
G_038	0.530	0.541	0.0109	Yes
G_039	0.521	0.448	−0.0733	No
G_040	0.278	0.276	−0.00199	No
G_041	0.620	0.470	−0.150	No
G_042	0.147	0.364	0.217	Yes

The score integrates VO₂ max and lactate measures. Δ CombinedEff represents the change from before to after the dietary intervention. Participants were classified as ‘Improved’ (Δ > 0) or ‘Not improved’ (Δ ≤ 0) based on the distribution of observed changes.

**Figure 7:**
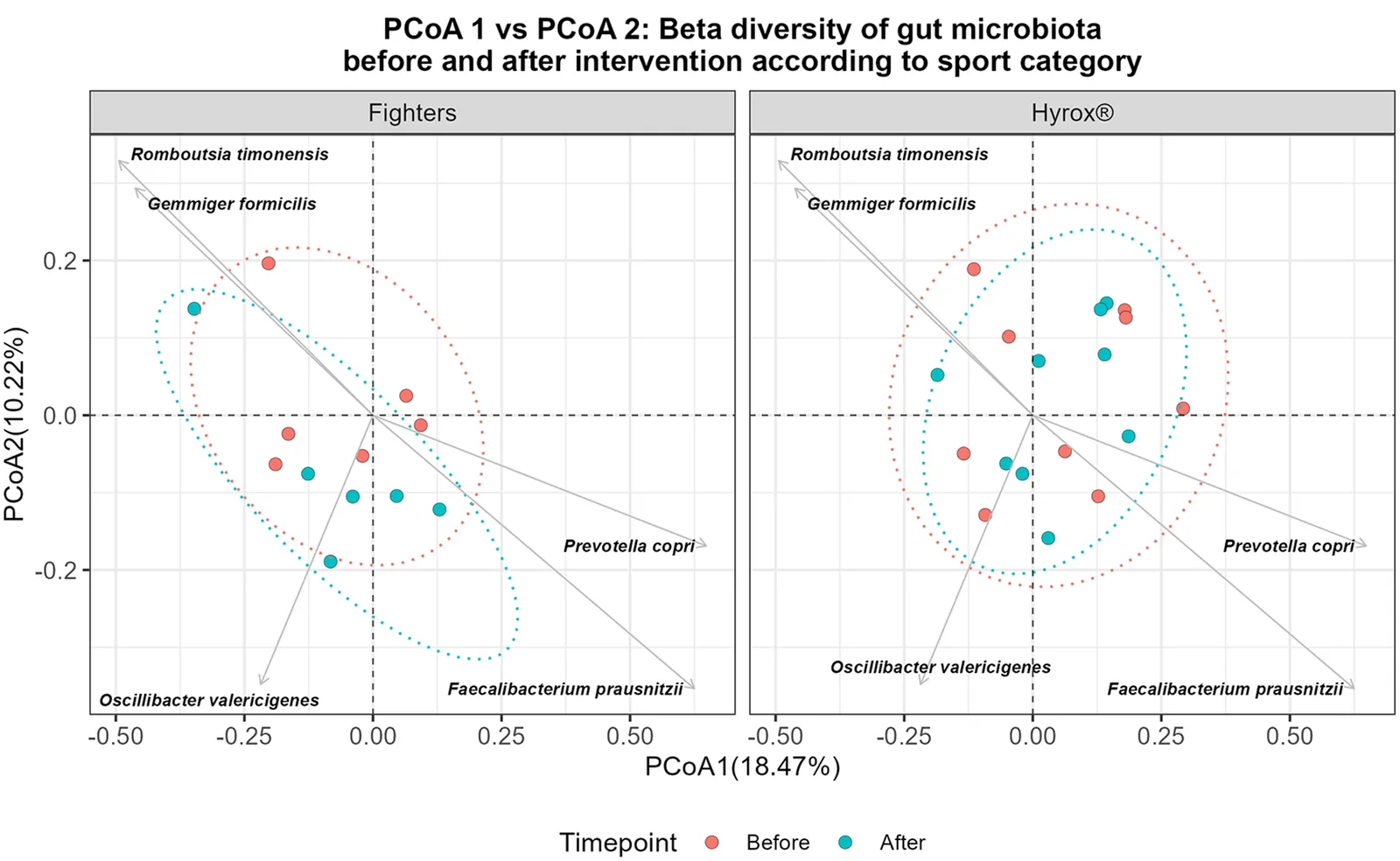
Principal coordinate analysis (PCoA) plots illustrating gut microbiota beta diversity (bray–curtis distance) at the species level, based on rarefied and hellinger-transformed OTU data. Sample points are colored by Timepoint (Before, red; After, blue) and faceted by Sport category (Fighters on the left, Hyrox® on the right). Ellipses represent 95% confidence intervals for each sport category. Selected species are displayed as arrows on the plot to indicate taxa that contribute most strongly to variation along the principal coordinates.

To assess whether changes in physiological performance were associated with differences in microbial diversity, linear mixed-effects models including a Timepoint×Improvement interaction were fitted for participants with available physiological data. No significant Timepoint×Improvement interaction was observed for any alpha-diversity metric (Observed richness: *F* = 0.95, *p* = 0.353; Shannon index: *F* = 0.47, *p* = 0.509; Simpson index: *F* = 0.86, *p* = 0.376), indicating that temporal changes in alpha diversity did not differ between participants classified as Improved and Not improved. The main effect of Improvement was significant only for Observed richness (*F* = 5.14, *p* = 0.047), whereas no significant effect was detected for the Shannon index (*F* = 0.20, *p* = 0.664) or Simpson index (*F* = 0.05, *p* = 0.826). Likewise, no significant main effect of Timepoint was observed for any alpha-diversity metric (Observed richness: *F* = 0.12, *p* = 0.732; Shannon index: *F* = 0.38, *p* = 0.552; Simpson index: *F* = 0.01, *p* = 0.910).These analyses did not detect significant differences in temporal alpha-diversity trajectories according to Improvement Status, although participants classified as Improved and Not improved differed in Observed richness irrespective of time. Collectively, these analyses did not detect statistically significant associations between alpha diversity and time, sport category, intervention type, or performance improvement status over the study period. Beta diversity was assessed using Bray–Curtis dissimilarity calculated on rarefied species-level abundance tables (Figure 7). To account for the paired structure of the data, all PERMANOVA tests were performed using adonis2 with 999 permutations constrained within individuals (strata=Participant ID).

First, we evaluated the presence of a global microbiota shift between timepoints across all subjects. No significant overall difference was observed between baseline and follow-up samples (PERMANOVA: *F* = 0.68, *R*^2^ = 0.0236, *p* = 0.17), indicating that no statistically significant temporal shift in gut microbial community structure was detected at the cohort level. We then investigated whether temporal changes were modulated by sport category using a marginal PERMANOVA model including interaction terms. The interaction between timepoint and sport category was not significant (*F* = 0.52, *R*^2^ = 0.0178, *p* = 0.53), suggesting that the absence of a global temporal shift was consistent across sport profiles. Homogeneity of group dispersions was verified using betadisper, showing no significant differences for timepoint (*p* = 0.49) or sport category (*p* = 0.32), confirming that group variances were comparable and supporting the validity of the PERMANOVA results.

To specifically explore whether physiological improvement was associated with changes in community structure, analyses were repeated on the subset of participants with available Improvement Status. In this subgroup, no significant shift between timepoints was observed (PERMANOVA: *F* = 0.72, *R*^2^ = 0.0317, *p* = 0.125), indicating that the present data did not provide evidence of a temporal shift in overall community structure. The Timepoint×Improvement interaction was not significant (PERMANOVA: *F* = 0.51, *R*^2^ = 0.0221, *p* = 0.571), indicating that temporal changes in beta diversity did not differ according to Improvement Status. Complementing these analyses, two sedentary individuals sampled at baseline and after three months without intervention exhibited minimal compositional drift (mean paired Bray–Curtis distance 0.29 ± 0.027) Because this observation is based on only two individuals, it should be interpreted descriptively and does not constitute evidence of microbiota stability in the absence of intervention. By comparison, Fighters and Hyrox participants showed slightly higher within-individual distances (0.37 ± 0.039 and 0.33 ± 0.033, respectively), reflecting modest microbiota changes under dietary or probiotic interventions.

Overall, these analyses did not detect statistically significant temporal changes in gut microbiota composition or significant differences in temporal dynamics according to sport category or performance improvement status over the study period. Differential abundance of individual taxa between timepoints was assessed using DESeq2 with a paired design, comparing Before vs. After within each subgroup. Analyses were performed separately for participants in the Hyrox® and Fighters sport categories, as well as for participants with available improvement data (Improvement = Yes/No). Only taxa with sufficient mean relative abundance (≥0.1%) were considered for downstream analyses. Following Benjamini–Hochberg correction for multiple testing, only taxa with adjusted *p*-values (padj < 0.05) were retained for visualization and interpretation.

**Figure 8:**
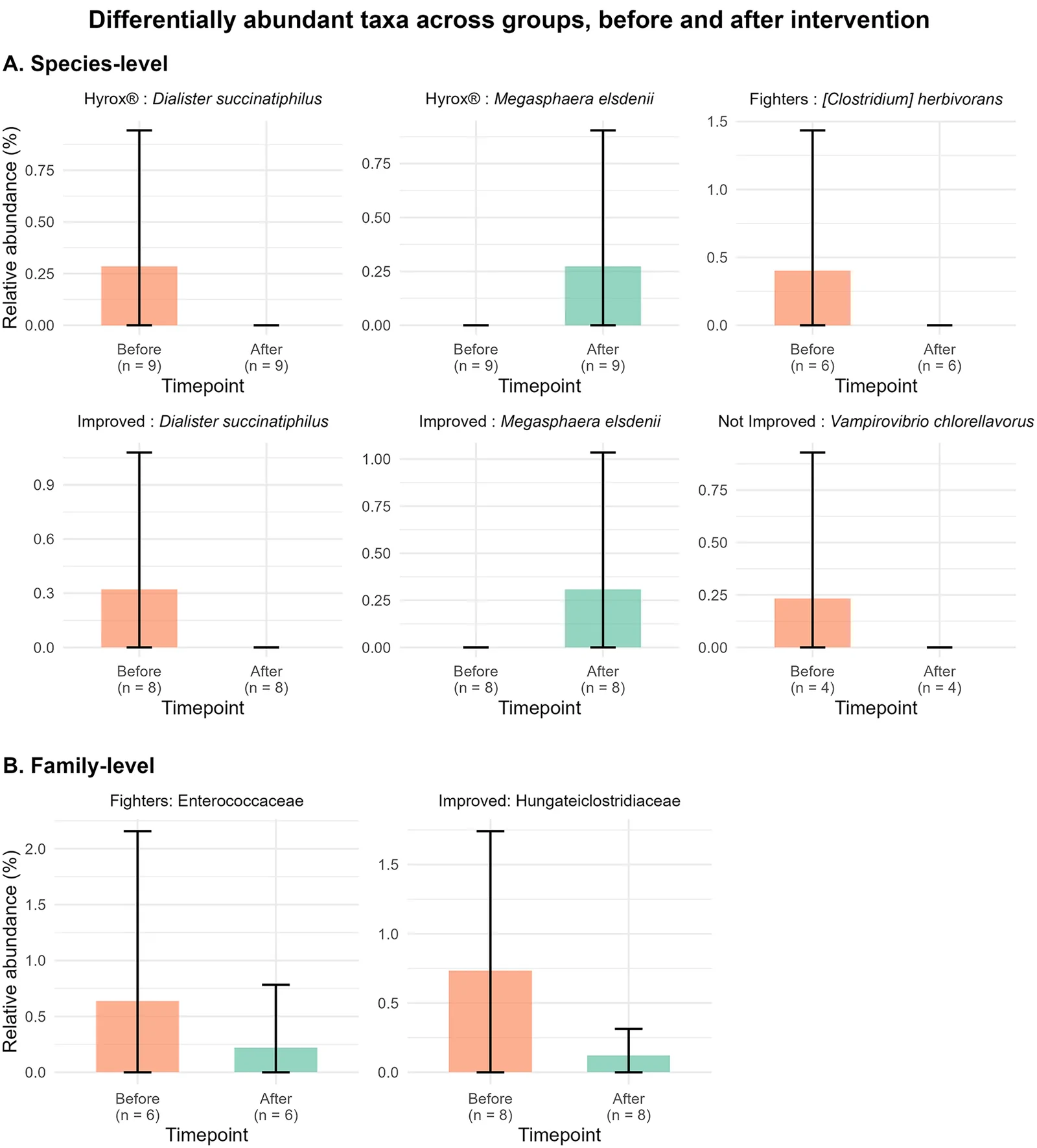
Relative abundance of gut microbiota taxa showing FDR-adjusted paired differences over the study period (p.adj < 0.05) across sport categories and improvement groups. Bars represent the mean relative abundance for each species **(A)** and family **(B)**, with error bars showing ± 95% confidence intervals.

At the species level, a limited number of taxa remained significantly different between baseline and follow-up after multiple-testing correction (Figure 8A). In Hyrox® participants, *Dialister succinatiphilus* decreased from 0.29% (95% CI: 0.00%–0.94%) before the intervention to undetectable levels after the intervention (0.00%, 95% CI: 0.00%–0.00%), whereas *Megasphaera elsdenii* increased from undetectable levels at baseline (0.00%, 95% CI: 0.00%–0.00%) to 0.27% (95% CI: 0.00%–0.90%) after the intervention. In Fighters, *Clostridium herbivorans* decreased from 0.40% (95% CI: 0.00%–1.43%) before the intervention to undetectable levels after the intervention (0.00%, 95% CI: 0.00%–0.00%). Among participants classified as Improved, *Megasphaera elsdenii* increased from undetectable levels at baseline (0.00%, 95% CI: 0.00%–0.00%) to 0.31% (95% CI: 0.00%–1.04%) after the intervention, while *Dialister succinatiphilus* decreased from 0.32% (95% CI: 0.00%–1.08%) to undetectable levels (0.00%, 95% CI: 0.00%–0.00%). Among participants classified as Not Improved, *Vampirovibrio chlorellavorus* decreased from 0.23% (95% CI: 0.00%–0.93%) before the intervention to undetectable levels after the intervention (0.00%, 95% CI: 0.00%–0.00%).At the family level, two microbial families remained significantly different after multiple-testing correction (Figure 8B). In Fighters, *Enterococcaceae* decreased from 0.64% (95% CI: 0.00%–2.16%) before the intervention to 0.22% (95% CI: 0.00%–0.78%) after the intervention. Among participants classified as Improved, *Hungateiclostridiaceae* decreased from 0.73% (95% CI: 0.00%–1.74%) at baseline to 0.12% (95% CI: 0.00%–0.31%) following the intervention. No significant family-level differences were observed among Hyrox® participants or participants classified as Not Improved. No statistically significant temporal differences were detected at the phylum level after multiple-testing correction.Because community-level analyses based on 16S rRNA gene sequencing did not detect statistically significant changes in gut microbiota diversity or community structure, we next applied shotgun metagenomic sequencing to explore whether functional changes occurred at the gene and pathway level. Shotgun metagenomic sequencing was performed on fecal samples from 9 Hyrox® athletes and 6 Fighters to compare the gut microbiota before and after intervention. The analysis focused on two main objectives: (i) to identify functional pathways assessed over the study period (diet or treatment) and (ii) to detect microbial genes associated with metabolism, SCFA production, and inflammation modulation.

**Figure 9:**
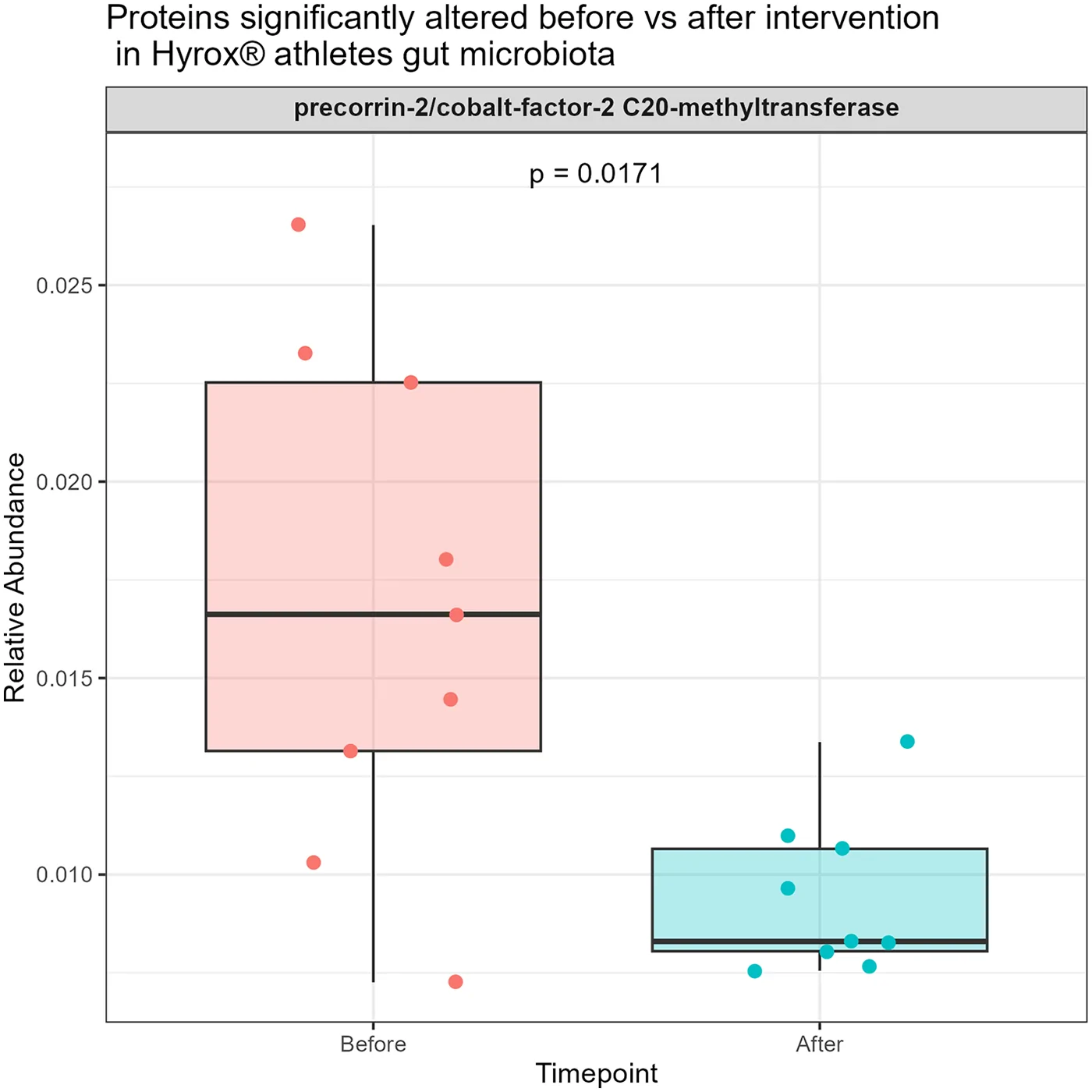
Relative abundance of gut microbial proteins showing nominally significant changes before and after intervention. Boxplots display paired sample values for each protein, with individual participants overlaid as points. Only precorrin-2/cobalt-precorrin-2 C20-methyltransferase is shown for Hyrox® athletes.

At the gene level, only one protein showed a nominally significant change in relative abundance in Hyrox® athletes (Figure 9): precorrin-2/cobalt-precorrin-2 C20-methyltransferase (median 0.0166 → 0.0083, *p* = 0.0171). This protein is functionally annotated to porphyrin and chlorophyll metabolism, suggesting a descriptive difference of this pathway following the intervention. After correction for multiple comparisons, the change in precorrin-2 in Hyrox® athletes was not statistically significant (adjusted *p* = 0.599). In Fighters, no proteins reached nominal significance (*p* < 0.05), indicating that no statistically significant gene-level changes were detected. However, the limited sample size may have reduced the ability to detect subtle functional differences.

**Figure 10:**
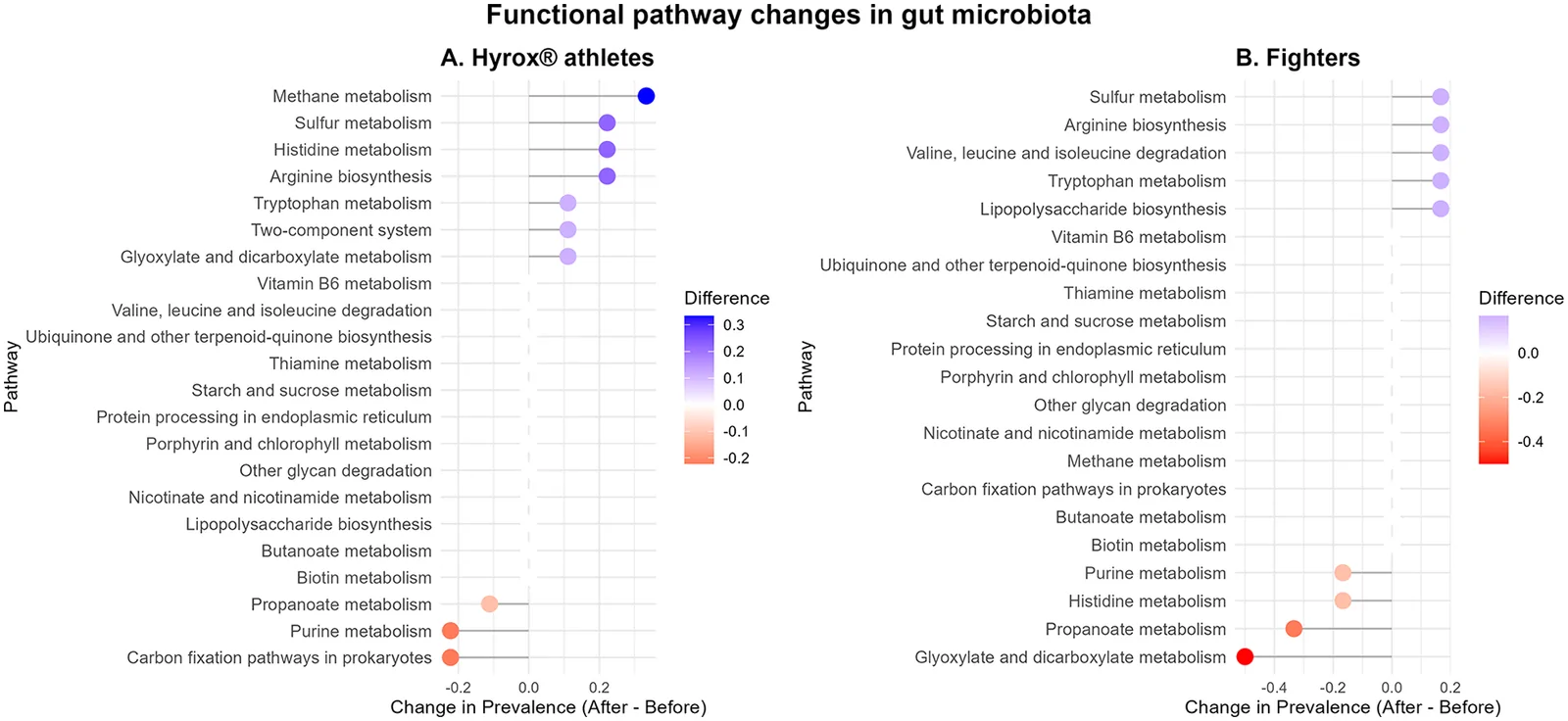
Lollipop plot showing the difference in prevalence of functional pathways after versus before intervention, in hyrox® athletes **(A)**, and in fighters **(B)** each point indicates the change in prevalence for a given pathway; red shows a decrease, blue an increase.

At the pathway level, we assessed 22 microbial functions in Hyrox® participants and 21 in Fighters (see Figure 10). Overall, no statistically significant pathway-level changes were detected between timepoints, although several descriptive differences in pathway prevalence were observed. In Hyrox®, nominal decreases were observed for carbon fixation pathways in prokaryotes (6/9 → 4/9 participants) and purine metabolism (8/9 → 6/9), while propanoate metabolism decreased from 1/9 to 0/9. Modest increases were observed for arginine biosynthesis, histidine metabolism, and sulfur metabolism (each increasing by 2 participants). Core pathways—including biotin metabolism, butanoate metabolism, nicotinate and nicotinamide metabolism, porphyrin and chlorophyll metabolism, and thiamine metabolism— were detected in all Hyrox® participants at both timepoints (Figure 10A).

In Fighters, subtle changes included decreases in glyoxylate and dicarboxylate metabolism (4/6 → 1/6) and propanoate metabolism (2/6 → 0/6), with small increases in arginine biosynthesis, lipopolysaccharide biosynthesis, valine/leucine/isoleucine degradation, and sulfur metabolism. Most core pathways remained unchanged (Figure 10B).

wStatistical analysis using McNemar tests indicated that none of these changes were statistically significant (*p* > 0.2), emphasizing that the observed shifts are descriptive and exploratory.

### 3.5 Taxa-specific associations with combined diet and prebiotic intervention in hybrid-sport athletes

**Figure 11:**
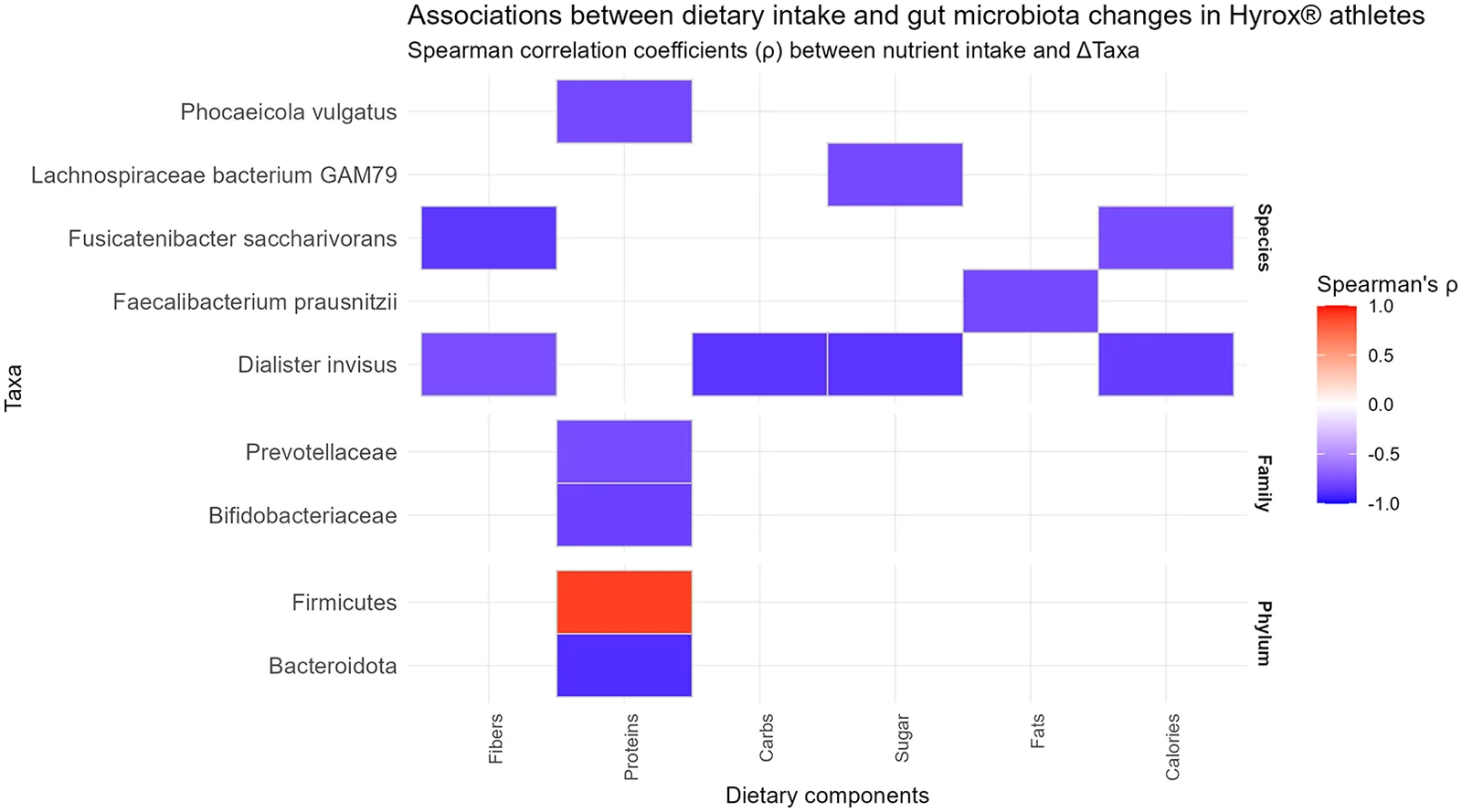
Heatmap showing exploratory associations between macronutrient intake during the intervention and changes in microbial taxa (ΔTaxa) in 9 paired hyrox® athletes. Only taxa with mean relative abundance >0.1% are included. Colors represent Spearman's correlation coefficients (*ρ*), with positive (red) and negative (blue) associations. Associations are shown for taxa reaching nominal significance (*p* < 0.05) Facets separate species, family, and phylum levels.

Hyrox® participants underwent a three-month intervention integrating personalized dietary adjustments and targeted prebiotic supplementation. These analyses included 9 paired Hyrox® participants and should therefore be interpreted cautiously. Analysis of paired changes in microbial taxa (ΔTaxa), considering only taxa with mean relative abundance >0.1%, identified several exploratory associations between nutrient intake and changes in microbial taxa. Benjamini–Hochberg correction was applied across all nutrient–taxon association tests; however, no associations remained statistically significant after false-discovery-rate correction. Therefore, the results presented below are based on nominal *p*-values (*p* < 0.05) and should be interpreted as exploratory observations (Figure 11).

At the species level, nominal exploratory associations were observed between macronutrient intake and specific taxa. For instance, *Fusicatenibacter saccharivorans* decreased with fiber intake (*ρ* = –0.86, *p* = 0.024) and with calories (*ρ* = –0.77, *p* = 0.041), while *Dialister invisus* showed negative correlations with fiber (*ρ* = –0.77, *p* = 0.044), carbohydrate (*ρ* = –0.87, *p* = 0.012), sugar (*ρ* = –0.87, *p* = 0.012), and calories (*ρ* = –0.84, *p* = 0.019). Protein intake was linked to reductions in *Phocaeicola vulgatus* (*ρ* = –0.79, *p* = 0.048) and the butyrate producer *Faecalibacterium prausnitzii* (*ρ* = –0.79, *p* = 0.048). Sugar intake also showed negative associations with *Lachnospiraceae bacterium* GAM79 (*ρ* = –0.79, *p* = 0.048). At the family level, *Prevotellaceae* (*ρ* = –0.77, *p* = 0.041) and *Bifidobacteriaceae* (*ρ* = –0.82, *p* = 0.034) decreased with protein intake, representing nominal associations observed across selected taxa. At the phylum level, *Bacteroidota* decreased (*ρ* = –0.89, *p* = 0.012) while *Firmicutes* increased (*ρ* =  + 0.89, *p* = 0.012) were associated with, indicating nominal phylum-level associations alongside species- and family-level changes. Because none of the nutrient–taxon associations remained statistically significant after Benjamini–Hochberg correction and the sample size was limited (*n* = 9), the associations presented here are based on nominal *p*-values and should be interpreted as exploratory observations.

No statistically significant associations were detected between prebiotic supplementation (GOS or FOS) and changes in microbial taxa in the present dataset. Overall, these exploratory analyses identified several nominal associations between macronutrient intake during the intervention and changes in the abundance of specific microbial taxa. Although exploratory associations were observed across multiple taxonomic levels, these findings should be regarded as hypothesis-generating and require validation in larger, adequately powered cohorts before biological conclusions can be drawn.

## 4 Discussion

In this exploratory cohort of 38 adults spanning sedentary, combat-trained, and hybrid-sport athletes, we investigated associations between study-group characteristics, short-term dietary/microbiota-targeted interventions and gut microbiota composition. No statistically significant differences in alpha diversity were detected over the 3-month intervention, suggesting that the present data did not provide evidence of marked temporal changes in overall microbial diversity. Nevertheless, fine-scale analyses identified study-group-associated differences in several microbial taxa: Fighters and Sedentary participants exhibited higher *Enterococcaceae* abundances relative to Hyrox® athletes, while *Dialister* *spp*. and *Succiniclasticum ruminis* showed differential abundance across sport categories. Paired differential-abundance analyses accounting for participant identity identified a limited number of taxa that remained significantly different after Benjamini–Hochberg correction for multiple testing. In Hyrox® participants, *Dialister succinatiphilus* decreased whereas *Megasphaera elsdenii* increased following the intervention. Among Fighters, *Clostridium herbivorans* decreased. Participants classified as “Improved” exhibited an increase in *Megasphaera elsdenii* together with a decrease in *Dialister succinatiphilus*, whereas participants classified as “Not improved” showed a decrease in *Vampirovibrio chlorellavorus*. Despite these taxon-specific differences, no statistically significant temporal changes in overall microbial diversity were detected, supporting the relative stability of the adult gut microbiota while suggesting that selected low-abundance taxa may exhibit context-dependent temporal variation.

The differences observed between participant groups are consistent with previous studies reporting associations between athletic status and gut microbiota composition. For example, Clarke et al. reported higher microbial diversity in professional rugby players compared with controls, correlating with protein intake and markers of intense exercise (3). Similarly, Humińska et al. showed that strength-trained athletes exhibited unique microbiome-biomarker associations following anaerobic exercise, while endurance training corresponded with baseline enrichment of taxa linked to performance responses (41). Supportive studies highlight the gut microbiota's role in modulating inflammation, energy metabolism, and oxidative stress during intense exercise, supporting the notion that specific taxa have been associated with exercise-related physiological phenotypes in previous studies (42, 43). These findings are consistent with previous reports describing associations between athletic populations and gut microbiota composition. However, because dietary intake, supplementation, training load, and other lifestyle factors differ across athletic populations, the independent contribution of exercise modality cannot be determined from the present study. Interestingly, the taxa that remained significant after multiple-testing correction were all relatively low-abundance organisms. *Megasphaera elsdenii* is a lactate-utilizing anaerobe involved in carbohydrate fermentation and the production of short-chain fatty acids, suggesting a potential role in microbial energy metabolism (44). In addition, *Dialister succinatiphilus* has previously been reported to differ between professional and amateur football players, with higher abundance in amateurs, further suggesting that this taxon may be associated with athletic status or training-related factors (45). However, because the present study was not designed to investigate microbial mechanisms, the biological significance of these observations remains uncertain and should be confirmed in larger longitudinal cohorts.

Shotgun metagenomic analyses provided complementary insight into the functional potential of the gut microbiome. Although descriptive differences were observed in several genes and pathways, including decreases in porphyrin-related methyltransferases in Hyrox® athletes and variations in pathways related to short-chain fatty acid (SCFA) metabolism and amino acid degradation, no statistically significant gene- or pathway-level differences were detected after correction for multiple testing. Previous studies have reported associations between exercise, diet and microbial metabolic functions related to energy metabolism, immune signalling, and mucosal integrity (6). However, given the limited size of the metagenomic subset, the inferential nature of sequencing-base functional profiling, and the exploratory scope of these analyses, the present findings do not allow conclusions regarding functional adaptation and require validation in larger, adequately powered studies using multi-omics techniques.

Methodologically, this study benefited from the inclusion of multiple athletic populations, paired within-subject sampling, and full-length 16S rRNA gene sequencing using Oxford Nanopore Technologies, enabling species-level taxonomic resolution beyond that achieved by short-amplicon sequencing. Integration of dietary records and physiological performance measurements provided additional context for interpreting the microbiome data. Nevertheless, several limitations should be acknowledged. First, this was a small, non-randomized, exploratory study, and the participant groups differed not only in exercise modality but also in dietary intervention and microbiota-targeted supplementation, limiting the ability to attribute observed associations to any individual component. Second, differential participant attrition between baseline and follow-up may have introduced selection bias, as some participants withdrew from the study or chose not to continue the intervention and therefore did not provide a follow-up fecal sample. Third, the shotgun metagenomic analysis was performed in a relatively small subset of participants (*n* = 15), reducing statistical power to infer subtle functional differences. Fourth, several subgroup analyses, including those based on Improvement Status, dietary intake, and taxonomic changes, were exploratory and should be interpreted accordingly. Although differential-abundance analyses were re-evaluated using paired statistical models accounting for repeated measurements and multiple-testing correction, the relatively small subgroup sizes limited statistical power, and independent validation in larger cohorts remains necessary. Future studies incorporating larger randomized cohorts, standardized dietary and supplementation protocols, metabolomic profiling, and metatranscriptomic analyses will be important to better characterize the functional relevance of the observed taxonomic associations. Collectively, the present exploratory findings identified study-group-associated differences in selected microbial taxa together with a limited number of within-group taxonomic changes that remained significant after paired differential-abundance analysis and correction for multiple testing. The beta-diversity and differential-abundance analyses together suggest that study-group characteristics may be associated with variation in specific microbial taxa, consistent with previous reports describing associations between athletic populations and gut microbiota composition (3, 41–43). However, these observations should not be interpreted as evidence that exercise modality independently shapes the gut microbiota, given the concurrent differences in dietary intervention and microbiota-targeted supplementation between study groups.

Overall, the present findings should be interpreted as observational and hypothesis-generating rather than evidence of causal effects. Future studies incorporating larger randomized cohorts, standardized dietary and supplementation protocols, and integrated multi-omics approaches such as metabolomics and metatranscriptomic analyses, will be necessary to better characterise the functional relevance of the observed taxonomic associations and to clarify the relationships between exercise, nutrition, and the gut microbiota. Ultimately, a better understanding of these interactions may help guide future research investigating personalised nutritional approaches to support athletic performance and long-term metabolic health.

## 5 Conclusion

This exploratory pilot study suggests that participation in distinct physical activity profiles, including hybrid sports that combine endurance and resistance training, may be associated with subtle fine-scale differences in gut microbiota composition. To our knowledge, athletes participating in hybrid sports have not been specifically examined in human gut microbiome studies. By directly comparing sedentary individuals, combat athletes, and hybrid-sport athletes, this study therefore extends exercise microbiome research beyond the endurance-focused paradigms that have dominated the field. While baseline microbial diversity and overall community structure were broadly comparable across groups, differential abundance analyses identified study-group-associated differences in selected microbial taxa at finer taxonomic resolution. Across the three-month observation period, no statistically significant large-scale changes in alpha diversity or beta diversity were detected, suggesting that the present study did not identify broad restructuring of gut microbial diversity over the observation period. Nevertheless, paired differential-abundance analyses accounting for repeated measurements identified a limited number of species- and family-level taxa that remained significant after Benjamini–Hochberg correction for multiple testing. These findings suggest subtle taxon-specific temporal changes that require validation in larger independent cohorts. Paired differential-abundance analyses identified a limited number of taxon-level differences over time within specific intervention groups after multiple-testing correction. Exploratory shotgun metagenomic analyses performed in a subset of paired samples did not identify statistically significant gene- or pathway-level differences after correction for multiple testing, although descriptive differences were observed in selected pathways., These observations should be interpreted cautiously and regarded primarily as hypothesis-generating given the limited sample size and exploratory nature of the analyses. Larger longitudinal studies integrating metabolomic profiling and deeper metagenomic resolution will be required to better characterize the relationship between exercise modality, nutrition, and gut microbiota composition and function.

## Data Availability

The datasets presented in this study can be found in online repositories. The names of the repository/repositories and accession number(s) can be found below: https://www.ebi.ac.uk/ena, PRJEB109268.

## References

[1] David LA Maurice CF Carmody RN Gootenberg DB Button JE Wolfe BE. Diet rapidly and reproducibly alters the human gut microbiome. Nature. (2014) 505:559–63. 10.1038/nature12820 24336217 PMC3957428

[2] Valdes AM Walter J Segal E Spector TD. Role of the gut microbiota in nutrition and health. Br Med J. [(2018) 361:k2179. 10.1136/bmj.k2179 29899036 PMC6000740

[3] Clarke SF Murphy EF O'Sullivan O Lucey AJ Humphreys M Hogan A. Exercise and associated dietary extremes impact on gut microbial diversity. Gut. (2014) 63:1913–20. 10.1136/gutjnl-2013-306541 25021423

[4] Barton W Penney NC Cronin O Garcia-Perez I Molloy MG Holmes E. The microbiome of professional athletes differs from that of more sedentary subjects in composition and particularly at the functional metabolic level. Gut. (2018) 67:625–33. 10.1136/gutjnl-2016-313627 28360096

[5] Scheiman J Luber JM Chavkin TA MacDonald T Tung A Pham L-D. Meta-omics analysis of elite athletes identifies a performance-enhancing microbe that functions via lactate metabolism. Nat Med. (2019) 25:1104–9. 10.1038/s41591-019-0485-4 31235964 PMC7368972

[6] Hughes RL Holscher HD. Fueling gut microbes: a review of the interaction between diet, exercise, and the gut Microbiota in athletes. Adv Nutri. (2021) 12:2190–215. 10.1093/advances/nmab077 PMC8634498 34229348

[7] Murach KA Bagley JR. Skeletal muscle hypertrophy with concurrent exercise training: contrary evidence for an interference effect. Sports Med. (2016) 46:1029–39. 10.1007/s40279-016-0496-y 26932769

[8] Franzosa EA McIver LJ Rahnavard G Thompson LR Schirmer M Weingart G. Species-level functional profiling of metagenomes and metatranscriptomes. Nat Methods. (2018) 15:962–8. 10.1038/s41592-018-0176-y 30377376 PMC6235447

[9] Visconti A Le Roy CI Rosa F Rossi N Martin TC Mohney RP. Interplay between the human gut microbiome and host metabolism. Nat Commun. (2019) 10:4505. 10.1038/s41467-019-12476-z 31582752 PMC6776654

[10] I S W G J K N A. Determinants of 2,000 m rowing ergometer performance in elite rowers. Eur J Appl Physiol. (2002) 88:243–6. 10.1007/s00421-002-0699-9 12458367

[11] Cosgrove MJ Wilson J Watt D Grant SF. The relationship between selected physiological variables of rowers and rowing performance as determined by a 2000m ergometer test. J Sports Sci. (1999) 17:845–52. 10.1080/026404199365407 10585164

[12] Concept2. VO2max Calculator for Indoor Rowing. *Concept2* Available online at: https://www.concept2.co.uk/training/vo2max-calculator (Accessed March 11, 2026)

[13] Hagerman FC Connors MC Gault JA Hagerman GR Polinski WJ. Energy expenditure during simulated rowing. J Appl Physiol. (1978) 45:87–93. 10.1152/jappl.1978.45.1.87 670038

[14] Beneke R Leithäuser RM Ochentel O. Blood lactate diagnostics in exercise testing and training. Int J Sports Physiol Perform. (2011) 6:8–24. 10.1123/ijspp.6.1.8 21487146

[15] Goodwin ML Harris JE Hernández A Gladden LB. Blood lactate measurements and analysis during exercise: a guide for clinicians. J Diabetes Sci Technol. (2007) 1:558–69. 10.1177/193229680700100414 19885119 PMC2769631

[16] Pinzauti D De Jaegher S D'Aguanno M Biazzo M. The human nasal microbiome: a perspective study during the SARS-CoV-2 pandemic in Malta. Microorganisms. (2024) 12:2570. 10.3390/microorganisms12122570 39770773 PMC11679825

[17] Pinzauti D Biazzo M Podrini C Alevizou A Safarika A Damoraki G. An NGS-assisted diagnostic workflow for culture-independent detection of bloodstream pathogens and prediction of antimicrobial resistances in sepsis. Front Cell Infect Microbiol. (2025) 15:1656171. 10.3389/fcimb.2025.1656171 40959141 PMC12434068

[18] De Coster W Rademakers R. Nanopack2: population-scale evaluation of long-read sequencing data. Bioinformatics. (2023) 39:btad311. 10.1093/bioinformatics/btad311 37171891 PMC10196664

[19] Martin M. Cutadapt removes adapter sequences from high-throughput sequencing reads. EMBnet J. (2011) 17:10. 10.14806/ej.17.1.200

[20] Callahan BJ McMurdie PJ Rosen MJ Han AW Johnson AJA Holmes SP. DADA2: high-resolution sample inference from illumina amplicon data. Nat Methods. (2016) 13:581–3. 10.1038/nmeth.3869 27214047 PMC4927377

[21] Li H. Minimap2: pairwise alignment for nucleotide sequences. Bioinformatics. (2018) 34:3094–100. 10.1093/bioinformatics/bty191 29750242 PMC6137996

[22] Marijon P Chikhi R Varré J-S. yacrd and fpa: upstream tools for long-read genome assembly. Bioinformatics. (2020) 36:3894–6. 10.1093/bioinformatics/btaa262 32315402

[23] Curry KD Wang Q Nute MG Tyshaieva A Reeves E Soriano S. Emu: species-level microbial community profiling of full-length 16S rRNA Oxford nanopore sequencing data. Nat Methods. (2022) 19:845–53. 10.1038/s41592-022-01520-4 35773532 PMC9939874

[24] Nurk S Koren S Rhie A Rautiainen M Bzikadze AV Mikheenko A. The complete sequence of a human genome. Science. (2022) 376:44–53. 10.1126/science.abj6987 35357919 PMC9186530

[25] Danecek P Bonfield JK Liddle J Marshall J Ohan V Pollard MO. Twelve years of SAMtools and BCFtools. GigaScience. (2021) 10:giab008. 10.1093/gigascience/giab008 33590861 PMC7931819

[26] Wood DE Lu J Langmead B. Improved metagenomic analysis with kraken 2. Genome Biol. (2019) 20:257. 10.1186/s13059-019-1891-0 31779668 PMC6883579

[27] Langmead B Kraken 2, KrakenUniq and Bracken indexes. Available online at: https://benlangmead.github.io/aws-indexes/k2 (Accessed February 20, 2026)

[28] Hyatt D Chen G-L LoCascio PF Land ML Larimer FW Hauser LJ. Prodigal: prokaryotic gene recognition and translation initiation site identification. BMC Bioinformatics. (2010) 11:119. 10.1186/1471-2105-11-119 20211023 PMC2848648

[29] Cantalapiedra CP Hernández-Plaza A Letunic I Bork P Huerta-Cepas J. eggNOG-mapper v2: functional annotation, orthology assignments, and domain prediction at the metagenomic scale. Mol Biol Evol. (2021) 12:5825–9 10.1101/2021.06.03.446934 PMC8662613 34597405

[30] Buchfink B Xie C Huson DH. Fast and sensitive protein alignment using DIAMOND. Nat Methods. (2015) 12:59–60. 10.1038/nmeth.3176 25402007

[31] Ye Y Doak TG. A parsimony approach to biological pathway reconstruction/inference for genomes and metagenomes. PLoS Comput Biol. (2009) 5:e1000465. 10.1371/journal.pcbi.1000465 19680427 PMC2714467

[32] R development Core Team. R: A Language and Environment for Statistical Computing: Reference index. Vienna: R Foundation for Statistical Computing. (2010).

[33] Posit team. R Studio: Integrated Development Environment for R. (2023) Available online at: http://www.posit.co/ (Accessed March 26, 2026).

[34] McMurdie PJ< Joey711@Gmail. Com > SHSE. Phyloseq (2017) 10.18129/B9.BIOC.PHYLOSEQ

[35] Lahti L Shetty S Microbiome R Package. (2017) 10.18129/B9.BIOC.MICROBIOME

[36] Wickham H Chang W Henry L Pedersen TL Takahashi K Wilke C. ggplot2: Create Elegant Data Visualisations Using the Grammarch of Graphics. (2007)3.5.1. 10.32614/CRAN.package.ggplot2

[37] Oksanen J Simpson GL Blanchet FG Kindt R Legendre P Minchin PR. Vegan: Community Ecology Package. (2001) 2.6-8. 10.32614/CRAN.package.vegan

[38] Michael Love SA. DESeq2. (2017) 10.18129/B9.BIOC.DESEQ2

[39] Wickham H Tidyverse: Easily Install and Load the “Tidyverse.” (2016) 2.0.0. 10.32614/CRAN.package.tidyverse

[40] Zierle-Ghosh A Jan A. “Physiology, Body Mass Index.,” StatPearls. Treasure Island (FL): StatPearls Publishing (2026) Available online at: http://www.ncbi.nlm.nih.gov/books/NBK535456/ [Accessed April 27, 2026) 30571077

[41] Humińska-Lisowska K Michałowska-Sawczyn M Kosciolek T Łabaj PP Kochanowicz A Mieszkowski J. Gut microbiome and blood biomarkers reveal differential responses to aerobic and anaerobic exercise in collegiate men of diverse training backgrounds. Sci Rep. (2025) 15:16061. 10.1038/s41598-025-99485-9 40341642 PMC12062308

[42] Mach N Fuster-Botella D. Endurance exercise and gut microbiota: a review. J Sport Health Sci. (2017) 6:179–97. 10.1016/j.jshs.2016.05.001 30356594 PMC6188999

[43] Mohr AE Jäger R Carpenter KC Kerksick CM Purpura M Townsend JR. The athletic gut microbiota. J Int Soc Sports Nutr. (2020) 17:24. 10.1186/s12970-020-00353-w 32398103 PMC7218537

[44] Shetty SA Marathe NP Lanjekar V Ranade D Shouche YS. Comparative genome analysis of Megasphaera sp. Reveals niche specialization and its potential role in the human gut. PLoS One. (2013) 8:e79353. 10.1371/journal.pone.0079353 24260205 PMC3832451

[45] Urban S Chmura O Wątor J Panek P Zapała B. The intensive physical activity causes changes in the composition of gut and oral microbiota. Sci Rep. (2024) 14:20858. 10.1038/s41598-024-71684-w 39242653 PMC11379964

[46] Fyfe JJ Bishop DJ Stepto NK. Interference between concurrent resistance and endurance exercise: molecular bases and the role of individual training variables. Sports Med. (2014) 44:743–62. 10.1007/s40279-014-0162-1 24728927

[47] Walsh NP Gleeson M Shephard RJ Gleeson M Woods JA Bishop NC. Position statement. Part one: immune function and exercise. Exerc Immunol Rev. (2011) 17:6–63. 21446352

